# TOP3A amplification and ATRX inactivation are mutually exclusive events in pediatric osteosarcomas using ALT

**DOI:** 10.15252/emmm.202215859

**Published:** 2022-08-03

**Authors:** Alexandre de Nonneville, Sébastien Salas, François Bertucci, Alexander P Sobinoff, José Adélaïde, Arnaud Guille, Pascal Finetti, Jane R Noble, Dimitri Churikov, Max Chaffanet, Elise Lavit, Hilda A Pickett, Corinne Bouvier, Daniel Birnbaum, Roger R Reddel, Vincent Géli

**Affiliations:** ^1^ Marseille Cancer Research Centre (CRCM), Inserm U1068, CNRS UMR7258, Institut Paoli‐Calmettes, Team « Telomere and Chromatin ». Equipe labellisée Ligue Nationale Contre Le Cancer Aix‐Marseille Univ Marseille France; ^2^ Cancer Research Unit, Faculty of Medicine and Health, Children's Medical Research Institute University of Sydney Westmead NSW Australia; ^3^ Predictive Oncology Laboratory, Marseille Cancer Research Centre (CRCM), Inserm U1068, CNRS UMR7258, Institut Paoli‐Calmettes Aix‐Marseille University Marseille France; ^4^ Department of Medical Oncology, CRCM, CNRS, INSERM, Institut Paoli‐Calmettes Aix‐Marseille Univ Marseille France; ^5^ Department of Medical Oncology Assistance Publique Hôpitaux de Marseille ‐ Timone Hospital Marseille France; ^6^ Telomere Length Regulation Unit, Faculty of Medicine and Health, Children's Medical Research Institute University of Sydney Westmead NSW Australia; ^7^ Department of Pathology Assistance Publique Hôpitaux de Marseille ‐ Timone Hospital Marseille France

**Keywords:** alternative lengthening of telomeres, ATRX, osteosarcomas, telomeres, TOP3A, Cancer, Chromatin, Transcription & Genomics, Musculoskeletal System

## Abstract

In some types of cancer, telomere length is maintained by the alternative lengthening of telomeres (ALT) mechanism. In many ALT cancers, the α‐thalassemia/mental retardation syndrome X‐linked (*ATRX*) gene is mutated leading to the conclusion that the ATRX complex represses ALT. Here, we report that most high‐grade pediatric osteosarcomas maintain their telomeres by ALT, and that the majority of these ALT tumors are ATRX wild‐type (wt) and instead carry an amplified 17p11.2 chromosomal region containing *TOP3A*. We found that *TOP3A* was overexpressed in the ALT‐positive ATRX‐wt tumors consistent with its amplification. We demonstrated the functional significance of these results by showing that TOP3A overexpression in ALT cancer cells countered ATRX‐mediated ALT inhibition and that TOP3A knockdown disrupted the ALT phenotype in ATRX‐wt cells. Moreover, we report that TOP3A is required for proper BLM localization and promotes ALT DNA synthesis in ALT cell lines. Collectively, our results identify TOP3A as a major ALT player and potential therapeutic target.

## Graphical abstract

There is an urgent need to treat pediatric osteosarcomas that present high frequency of alternative lengthening of telomeres (ALT). This study identifies TOP3A amplification as mutually exclusive with ATRX inactivation, promoting ALT and TOP3A action at telomeres as a novel therapeutic target.

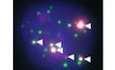

## Introduction

The vast majority of human cancers upregulate telomerase, while the others rely on a mechanism called alternative lengthening of telomeres (ALT) based on homologous recombination (HR)‐mediated DNA replication (Sobinoff & Pickett, 2020). The choice between telomerase and ALT seems dependent on the cell‐of‐origin of the tumor (Lafferty‐Whyte *et al*, 2009; Claude & Decottignies, 2020). Tumors originating from epithelial cells endowed with low telomerase activity frequently upregulate telomerase through mutations in the *TERT* promoter (Chiba *et al*, 2017) or genomic rearrangements (Peifer *et al*, 2015). In tumors of mesenchymal or neuroectodermal origin, activation of TERT expression is less common and these tumors exhibit a high frequency of ALT activation (Kent *et al*, 2020).

Alternative lengthening of telomeres is characterized by specific features including heterogeneous telomere length, partial clustering of telomeric DNA in promyelocytic leukemia (PML) bodies, partially double‐stranded circles with an intact C‐strand (C‐circles), and telomere sister chromatid exchanges (Yeager *et al*, 1999; Cesare & Griffith, 2004; Londoño‐Vallejo *et al*, 2004; Henson *et al*, 2009), although this last feature cannot be assessed directly on tumor tissue. It is accepted that tumors that acquire the ALT phenotype are prone to replication stress, which when unresolved may lead to fork collapse and as a consequence to double‐strand breaks (DSBs). ALT uses these breaks to initiate break‐induced telomere synthesis by RAD51‐dependent and RAD52‐dependent mechanisms (Verma *et al*, 2019; Zhang *et al*, 2019; Hoang & O'Sullivan, 2020). Multiple genomic repair pathways converge at telomeres to promote replication fork restart and telomere break repair (Sobinoff *et al*, 2017). Disruption of telomere chromatin may be at the root of replicative stress (Jiang *et al*, 2011; O'Sullivan *et al*, 2014). Recent results indicate that telomeres can be also elongated during mitosis (mitotic DNA synthesis) in ALT‐associated PML body (APB)‐like foci to counteract replication stress (Garcia‐Exposito *et al*, 2016; Min *et al*, 2017; Özer & Hickson, 2018).

The α‐thalassemia/mental retardation syndrome X‐linked (ATRX) protein is a SWI/SNF2‐like chromatin remodeler that deposits histone H3.3 at pericentric heterochromatin and telomeres (Goldberg *et al*, 2010; Wong *et al*, 2010). *ATRX*, or the death domain‐associated protein (*DAXX*) gene which encodes a protein that forms a heterodimeric complex with ATRX, is frequently inactivated by mutation in tumors with ALT (Heaphy *et al*, 2011; Lovejoy *et al*, 2012). Because ATRX limits replication stress (Clynes *et al*, 2013; Leung *et al*, 2013; Huh *et al*, 2016) and telomeres are prone to replication stress (Sfeir *et al*, 2009), loss of ATRX likely results in telomeric DSBs that initiate homology‐directed repair at telomeres (Hoang *et al*, 2020; Sobinoff & Pickett, 2020). In human tumors, ALT was described as associated with mutations in the ATRX pathway (Heaphy *et al*, 2011). This is well established in ALT‐positive gliomas in which the IDH1^R132H^ mutation is associated with ATRX loss (Nguyen *et al*, 2013; Ohba *et al*, 2020). ATRX loss has been proposed as a marker of ALT (Sieverling *et al*, 2020), but the view that ATRX/DAXX loss is essentially equivalent to the presence of ALT activity may apply only to specific types of tumors, in specific genomic or epigenetic contexts, and may divert attention from the need to identify alternative molecular actors involved in this telomere maintenance mechanism (TMM; Barthel *et al*, 2017; Brosnan‐Cashman *et al*, 2018; Graham *et al*, 2019; de Nonneville & Reddel, 2021).

High‐grade osteosarcomas are highly aggressive malignant bone tumors that mainly occur in children and adolescents. Their therapeutic management includes neoadjuvant chemotherapy, which in pediatric contexts consists of the combination of high‐dose methotrexate, ifosfamide, and VP16, followed by surgery, and then adjuvant chemotherapy (Le Deley *et al*, 2007). When metastatic, the 5‐year survival rate of these tumors is less than 30%, indicating the critical need for new treatment strategies (Sayles *et al*, 2019). Osteosarcomas exhibit a high frequency of ALT activation (Ulaner *et al*, 2003; Sanders *et al*, 2004; Henson *et al*, 2005). Strikingly, previous reports showed that *ATRX* mutation and/or loss of protein expression is detectable in only 30% of ALT‐positive osteosarcomas (Chen *et al*, 2014; Liau *et al*, 2015). This discrepancy between a high level of ALT and a low proportion of ATRX inactivation led us to hypothesize that ATRX alteration may not be the only alteration responsible for the ALT mechanism in osteosarcoma.

We have here analyzed the TMM in 22 non‐metastatic, non‐pre‐treated, high‐grade pediatric osteosarcomas. We identified *TOP3A* amplification and overexpression as a mutually exclusive event with ATRX inactivation, and validated TOP3A as an ALT regulator in the *ATRX* wild‐type (wt) setting. We also identified a number of genes for which mutation, amplification, or loss of copy number are closely associated with ALT, revealing new insights into the mechanisms by which telomeres are maintained. Moreover, our data suggest that ALT‐positive osteosarcomas may represent different entities in term of therapeutic opportunities.

## Results

### High frequency of ALT in high‐grade osteosarcomas

**Figure 1:**
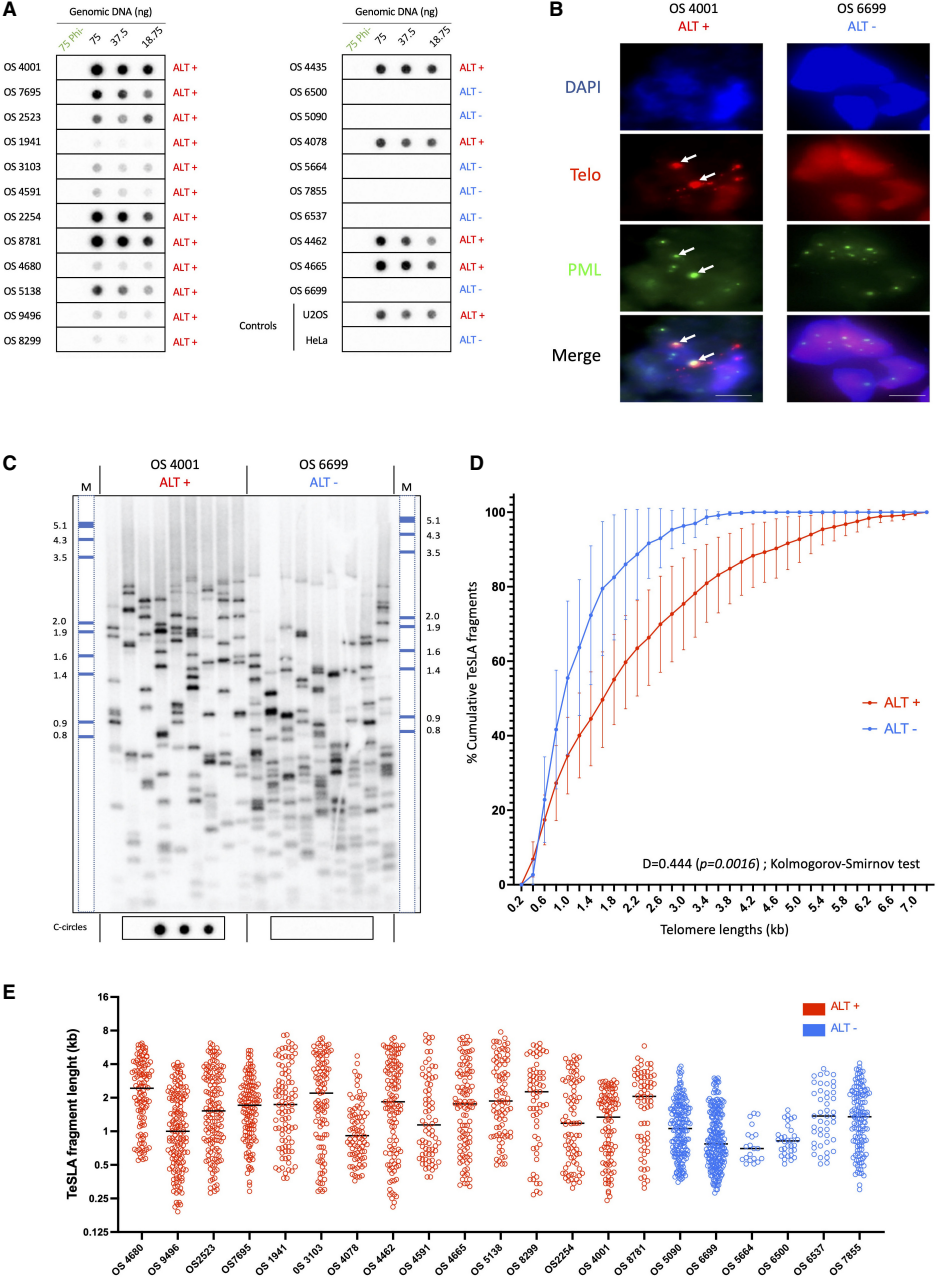
High frequency of alternative lengthening of telomeres (ALT) in high‐grade osteosarcomas C‐circle assay for tumor samples. The presence of C‐circles was tested in 22 osteosarcomas by Rolling Circle Amplification (RCA) assay using Φ29 DNA polymerase. Φ29 DNA polymerase negative controls and Φ29 DNA polymerase‐based reactions starting, respectively, with 75, 37.5, and 18.75 ng of DNA were applied to dot blots and C‐circles were detected by hybridization with a ^32^P‐(CCCTAA)_4_ telomeric probe. U2OS and HeLa cells correspond to positive and negative control samples, respectively.Representative images of telomere fluorescence *in‐situ* hybridization (FISH; red) and promyelocytic leukemia (PML) immunofluorescence IF (green) colocalizations (ALT‐associated PML body [APBs]) in osteosarcoma tumor samples. DNA is counterstained blue with DAPI. APBs are indicated by white arrows. Scale bars are 5 μm.Representative image of telomere shortest length assay (TeSLA) Southern Blot of two representative samples of ALT‐positive (left) and telomerase‐positive (right) osteosarcomas. Nine TeSLA polymerase chain reactions (PCRs; 30 pg each reaction) were done for each DNA sample. DIG‐labeled MW ladder has been added a posteriori.Plot of cumulative TeSLA fragment sizes (in kb) in ALT‐positive (*n* = 16) and ALT‐negative (*n* = 6) osteosarcomas. Points and error bars (±SEM) represent cumulative percentage of TeSLA fragments from all the TeSLA PCRs (*n* = 9 per tumor sample) from all the patients in both groups (*n* = 16 in ALT‐positive group, and *n* = 6 in ALT‐negative group). The Kolmogorov–Smirnov test was applied to identify statistical differences in TeSLA fragments distributions (*P* = 0.0016).Individual TeSLa fragment lengths in ALT‐positive (*n* = 16) and ALT‐negative (*n* = 6) osteosarcomas. Each dot represents a TeSLA fragment, bars represent the median value for individual patient's samples.

We characterized a series of high‐grade osteosarcomas from 22 non‐metastatic and non‐pre‐treated pediatric patients whose clinico‐pathological characteristics are described in Table 1. Using the presence of circular partially single‐stranded extrachromosomal C‐rich telomeric repeat sequences (C‐Circles) as an ALT marker, we found that 16 of the 22 high‐grade osteosarcomas were ALT positive (Fig 1A). The C‐circle assay‐based classification was consistent with the presence or absence of APBs in ALT‐positive and ‐negative tumors, respectively (Fig 1B shows a representative photomicrograph; APB analysis was done on 14/22 tumors due to limited availability of tumor material). To explore telomere length distribution in the tumor samples, we further used a telomere shortest length assay (TeSLA), which allows the evaluation of the size of individual telomeres in the bulk population of telomeres (Lai *et al*, 2017). In ALT‐positive tumors, telomere length was more heterogeneous with an increase in the median telomeric fragment size consistent with the general organization of ALT telomeres (Cesare & Reddel, 2010; Fig 1C–E). Thus, by using three different criteria, we were able to unambiguously determine that 73% of our series of high‐grade osteosarcomas maintained their telomeres by ALT.

**Table 1:** Clinico‐pathological characteristics of 22 pediatric osteosarcomas.

Sex	Age at diagnosis	Topography	Histology	Tumor size (mm)	Pathological response to neoadjuvant chemotherapy	ALT
Female	16	Proximal femoral diaphysis	Osteoblastic and chondroblastic	22	Good	ALT neg
Female	11	Mid‐tibial diaphysis	Chondroblastic		Good	ALT neg
Female	9	Proximal tibial diaphysis	Conventional	76	Good	ALT pos
Female	14	Tibial metaphysis	Conventional	82	Good	ALT pos
Female	13	Femoral diaphysis			Good	ALT pos
Female	5	Proximal femoral metaphysis	Osteogenic	110	Good	ALT pos
Female	12	Distal femoral diaphysis	Osteoblastic and chondroblastic	180	Good	ALT pos
Male	14	Distal femoral diaphysis	Osteoblastic and chondroblastic	34	Good	ALT neg
Male	14	Proximal humeral diaphysis		174	Good	ALT pos
Male	8	Distal femoral metaphysis	Osteoblastic	140	Good	ALT pos
Male	17	Proximal humeral diaphysis	Osteoblastic and chondroblastic	150	Good	ALT pos
Male	10	Proximal tibial metaphysis	Osteogenic	95	Good	ALT pos
Male	14	Proximal humeral diaphysis	Osteoblastic	155	Good	ALT neg
Female	15	Distal femoral diaphysis	Osteoblastic		Poor	ALT neg
Female	10	Distal femoral diaphysis	Osteoblastic	75	Poor	ALT pos
Female	17	Proximal tibial diaphysis	Osteoblastic	112	Poor	ALT pos
Male	19	Distal femoral metaphysis		100	Poor	ALT neg
Male	14	Femoral diaphysis	Osteoblastic		Poor	ALT pos
Male	15	Distal femoral diaphysis	Osteogenic	80	Poor	ALT pos
Male	13	Distal femoral diaphysis	Chondroblastic	90	Poor	ALT pos
Male	13	Proximal tibial diaphysis	Chondroblastic	100	Poor	ALT pos
Male	17	Distal femoral diaphysis	Chondroblastic	210	Poor	ALT pos

ALT, alternative lengthening of telomeres.

**Figure EV1:**
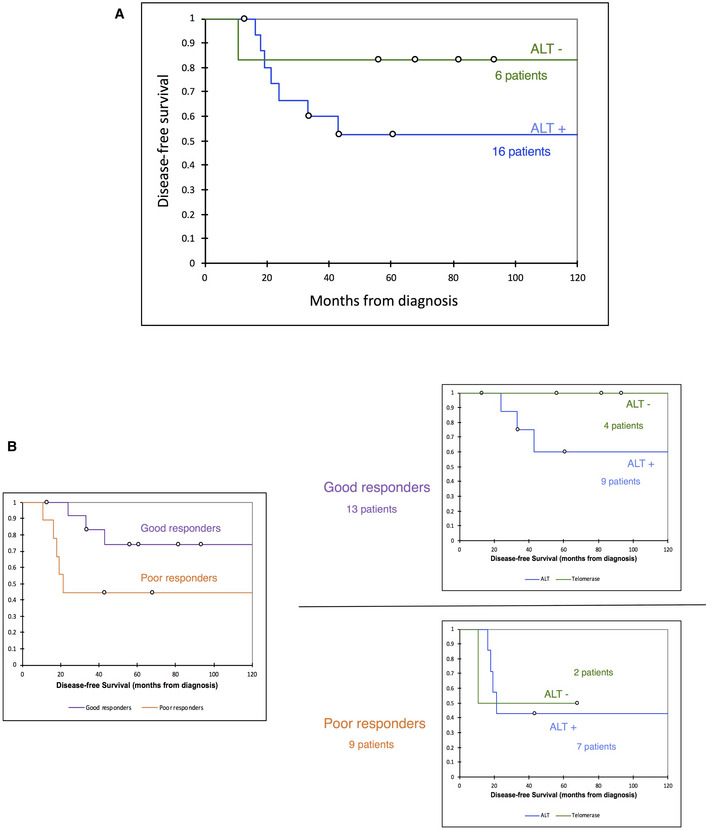
Survival analysis according to ALT Disease‐free survival curves of patients with ALT‐positive and ALT‐negative tumors.Disease‐free survival curves of good and poor responders to neoadjuvant chemotherapy (left panel), then dichotomized according to ALT status of tumors (right panel).

Compared to patients with ALT‐negative tumors, patients with ALT‐positive tumors had no significant differences regarding age at diagnosis, sex, tumor size, response to neoadjuvant chemotherapy, relapse rate, or deaths (Table 2). With a median follow‐up of 126.2 months, 7 out of 16 patients with an ALT‐positive tumor, and 1 out of 6 patients with an ALT‐negative tumor relapsed. The 5‐year Disease‐Free Survival (DFS) was 55.6% in ALT‐positive tumors vs. 83.3% in ALT‐negative tumors, but the difference was not significant likely because of the small cohort size (*P* = 0.319; Log‐rank test; Fig EV1A). When the cohort was stratified according to the pathological response to neoadjuvant chemotherapy, poor responders, whether ALT positive or ALT negative, experienced poor DFS outcomes, whereas in the good responders group, only ALT‐positive patients relapsed with 64.8% 5‐year DFS vs. 100% in ALT‐negative patients (*P* = 0.205; Log‐rank test; Fig EV1B).

**Table 2:** Correlation between alternative lengthening of telomeres (ALT) status and clinico‐pathological characteristics.

	All		ALT‐negative		ALT‐positive		*P*‐value (Khi2)
	(*n* = 22)		(*n* = 6)		(*n* = 16)		
	No. (%)		No. (%)		No. (%)		
Age at diagnosis (years)							
Median [Min–Max]	14 [5–19]		14.5 [11–19]		13 [5–17]		0.159
Sex							
Male	12	55%	3	50%	9	56%	0.793
Female	10	45%	3	50%	7	44%	
Radiological size in mm (mean, Std. Deviation)	110.3, 49.7		77.8, 61.9		119.6, 43.9		0.142
Pathological response to neoadjuvant chemotherapy							
Poor responder	9	41%	2	33%	7	44%	0.658
Good responder	13	59%	4	67%	9	56%	
Metastatic relapse							
No	14	64%	5	83%	9	56%	0.24
Yes	8	36%	1	17%	7	44%	
Death							
No	16	73%	5	83%	11	69%	0.494
Yes	6	27%	1	17%	5	31%	

**Figure 2:**
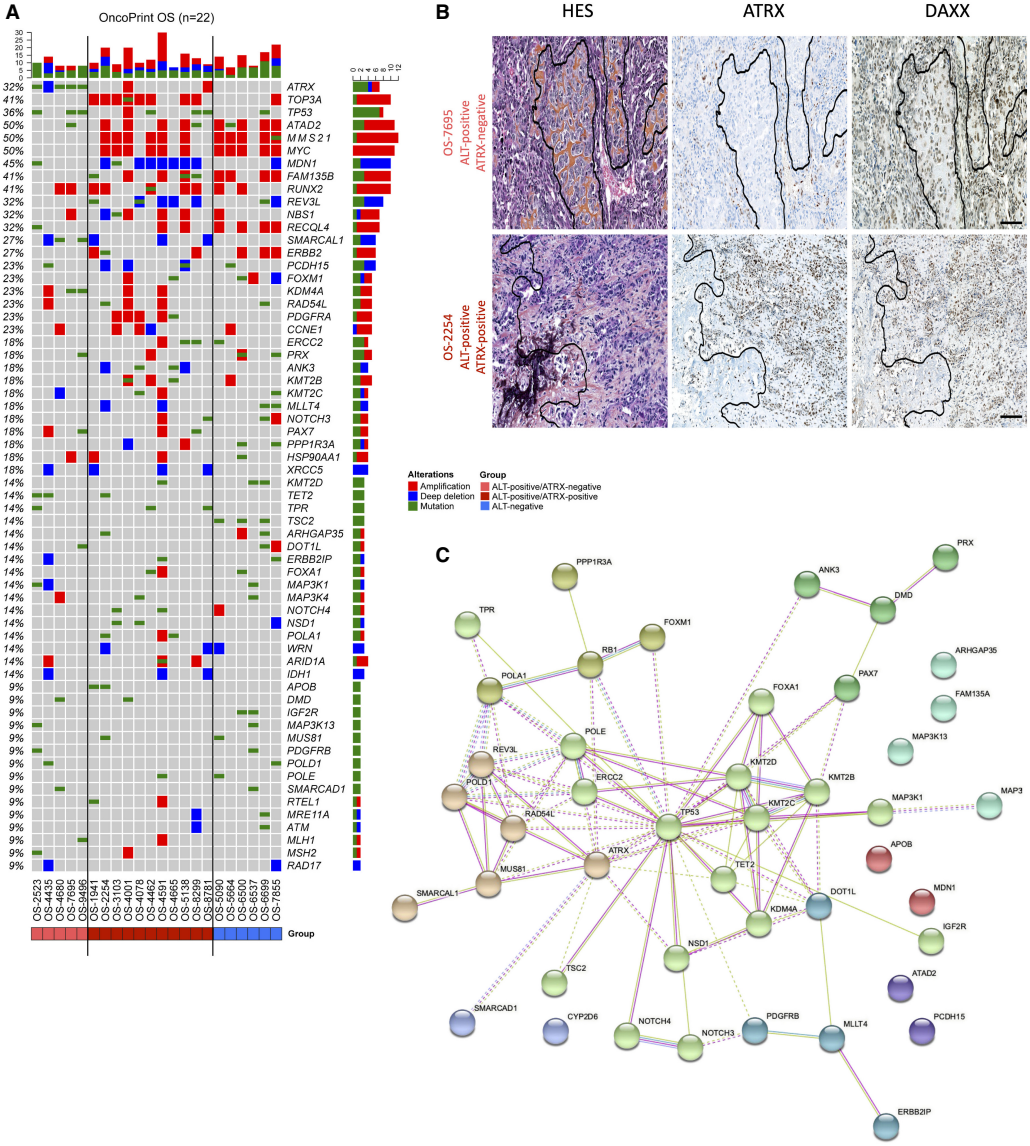
α‐thalassemia/mental retardation syndrome X‐linked wild‐type (ATRX‐wt) in expressed in alternative lengthening of telomeres (ALT)‐positive high‐grade osteosarcomas Oncoprint graph showing the distribution of mutations (green rectangles) and copy number variations (red rectangle, amplification; blue rectangle, copy loss) in all samples. Tumors are distributed according to ALT positivity (ALT^+^) or ALT negativity (ALT^−^) and *ATRX* status (ATRX‐mutated or ATRX‐wt).Tissue sections of two representative high‐grade osteosarcomas with hematoxylin eosin saffron (HES) staining (left panel), anti‐ATRX (HPA064684) immunochemistry (middle panel), and anti‐DAXX (HPA008736) immunochemistry (right panel), showing ATRX protein expression in two ALT‐positive samples. In the top panel, intratumoral ATRX expression is negative (positive control osteoclasts are shown). In the bottom panel, intratumoral ATRX expression is high. Scale bars are 250 μm.STRING network showing mutated gene‐clusters with related functions.

### ALT‐positive high‐grade osteosarcomas mostly express wt ATRX

**Figure EV2:**
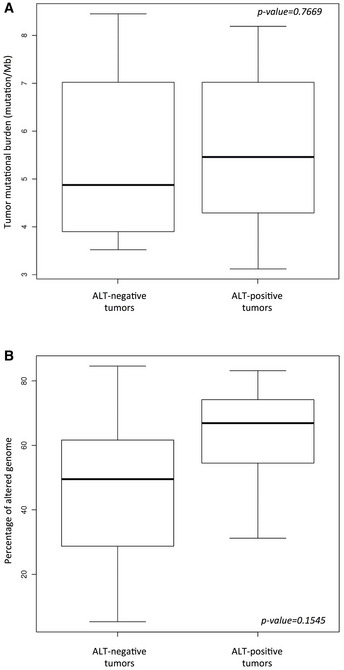
No difference was observed in tumor mutational burden or percentage of altered genome according to ALT Tumor mutational burden in ALT‐negative (*n* = 6) and ALT‐positive (*n* = 16) tumors. Box‐and‐whisker plot were defined with default parameters by median value (central band at the 50^th^ percentile), interquartile ranges (IQR, box limited by 25^th^ and 75^th^ percentile) and whisker boundaries defined by minimum and maximum value. Wilcoxon test was used to compare modalities.Percentage of altered genome using array‐comparative genomic hybridization (aCGH) in ALT‐negative (*n* = 6) and ALT‐positive (*n* = 16) tumors. Box‐and‐whisker plot were defined with default parameters by median value (central band at the 50^th^ percentile), interquartile ranges (IQR, box limited by 25^th^ and 75^th^ percentile) and whisker boundaries defined by minimum and maximum value. Wilcoxon test was used to compare modalities.

We next applied targeted next‐generation sequencing (tNGS) to the 22 tumors using a panel of 756 genes (Table EV1). With an average sequencing depth of 994×, we identified in the whole series a total of 288 mutations in 200 different genes the potential function of which in telomere maintenance is indicated in Table EV2. We found 244 single‐nucleotide variants (non‐synonymous and stop‐gains), 25 indels, and 1 splice‐site mutation (Table EV2). The median mutational burden per tumor (5.46) was higher in ALT‐positive tumors than in ALT‐negative tumors, but the difference was not significant (Fig EV2).

tNGS revealed recurrent genetic alterations (7/22) in *TP53* as previously reported (Chen *et al*, 2014; Kovac *et al*, 2015; Bousquet *et al*, 2016), with 6 out of the 7 mutated tumors being in the ALT‐positive group (Fig 2A, Table EV2). Overall, 13 genes were found mutated in at least three tumors (*TP53, ATRX, ATAD2, ERCC2, FAMA35B, KMT2D, PCH15, PRX, RB1, REV3L, TET2, TPR*, and *TSC2*, the functions of which are described in Table EV1). Strikingly, only 4 out of the 16 ALT‐positive tumors had an *ATRX* mutation and 1 of the 16 had an *ATRX* deep deletion (Fig 2A; see also the array‐comparative genomic hybridization [aCGH] data). Moreover, none of the tumors exhibited a mutation in the gene encoding the ATRX‐associated protein DAXX. Consistent with the tNGS results, immunohistochemistry analysis of 14/22 available tumors confirmed ATRX and DAXX protein expression in those identified by DNA sequencing as wt for *ATRX* and *DAXX* (Fig 2B). We concluded that 69% (11/16) of the ALT‐positive tumors displayed a seemingly unaltered ATRX/DAXX complex. Our results reveal that a majority of pediatric osteosarcomas were able to maintain their telomeres by ALT while expressing wt ATRX.

Many of the mutated genes were functionally related (Fig 2C and Table EV1). For instance, in addition to *ATRX*, several play a role in replication, replication stress response, or DNA repair (*MUS81, ERCC2, SMARCAD1, SMARCAL1, POLA1, POLE, POLD1, REV3L*, and *RAD54L*). Along the same line, although the frequency of mutation for each individual gene was low, 16 of the 22 tumors were mutated in genes associated with histone lysine methylation (*KMT2B, KMT2C, KMT2D, DOT1, NSD1, KDM4A*, and *TET2*) or nucleosome remodeling (*SMARCAD1*; Fig 2C and Table EV1). Although most of these genes were not previously reported to be altered in osteosarcoma including pediatric forms (Chen *et al*, 2014; Kovac *et al*, 2015; Bousquet *et al*, 2016; Sayles *et al*, 2019), and although the mutations detected were not functionally validated, our results suggest that the epigenetic landscape in these pediatric tumors might be frequently altered (see Discussion).

**Figure EV3:**
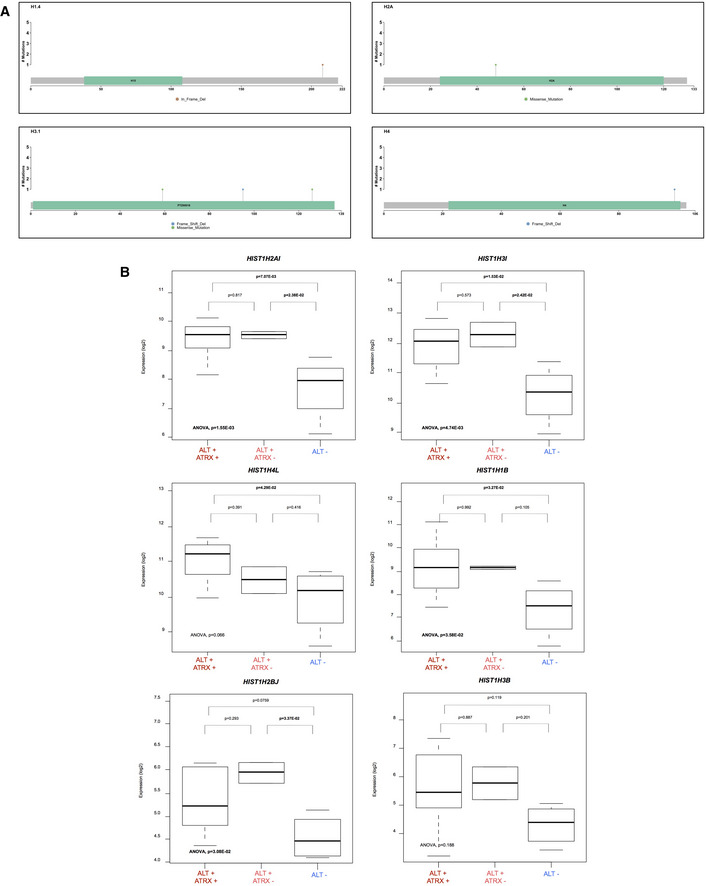
Histone genes in patient's osteosarcoma tumors Lollipop plots of *H1.4*, *H2A*, *H3.1*, and *H4* genes showing histone mutations detected by tNGS.Expression of histone genes according to ALT and ATRX status: ALT^+^/ATRX^+^ (*n* = 7), ALT^+^/ATRX^−^ (*n* = 2) et ALT^−^ (*n* = 3). Box‐and‐whisker plot were defined with default parameters by median value (central band at the 50^th^ percentile), and interquartile ranges (IQR, box limited by 25^th^ and 75^th^ percentile) and whisker boundaries were defined by minimum and maximum value. An ANOVA statistical test, and Tukey's range test were used to compare modalities.

We also sequenced canonical histone genes of the *HIST1* cluster that are frequently mutated in sarcomas (Nacev *et al*, 2019; Flaus *et al*, 2021). We found histone mutations in *HIST1H1B, HIST1H1C, HIST1H1D, HIST1H1E, HIST1H1T, HIST1H2AJ, HIST1H2AM, HIST1H2BF, HIST1H3A, HIST1H3B, HIST1H4H*, and *HIST1H4L* potentially expanding the landscape of oncohistone mutations in osteosarcoma (Table EV2 and Fig EV3; Nacev *et al*, 2020). Of note, 11/13 of the histone mutations including two mutations in *HIST1H3A* and *HIST1H4H* that modify their C‐terminal region were found in ALT‐positive tumors suggesting that these different mutations may contribute to a favorable environment for ALT activity (see Discussion).

**Figure 3:**
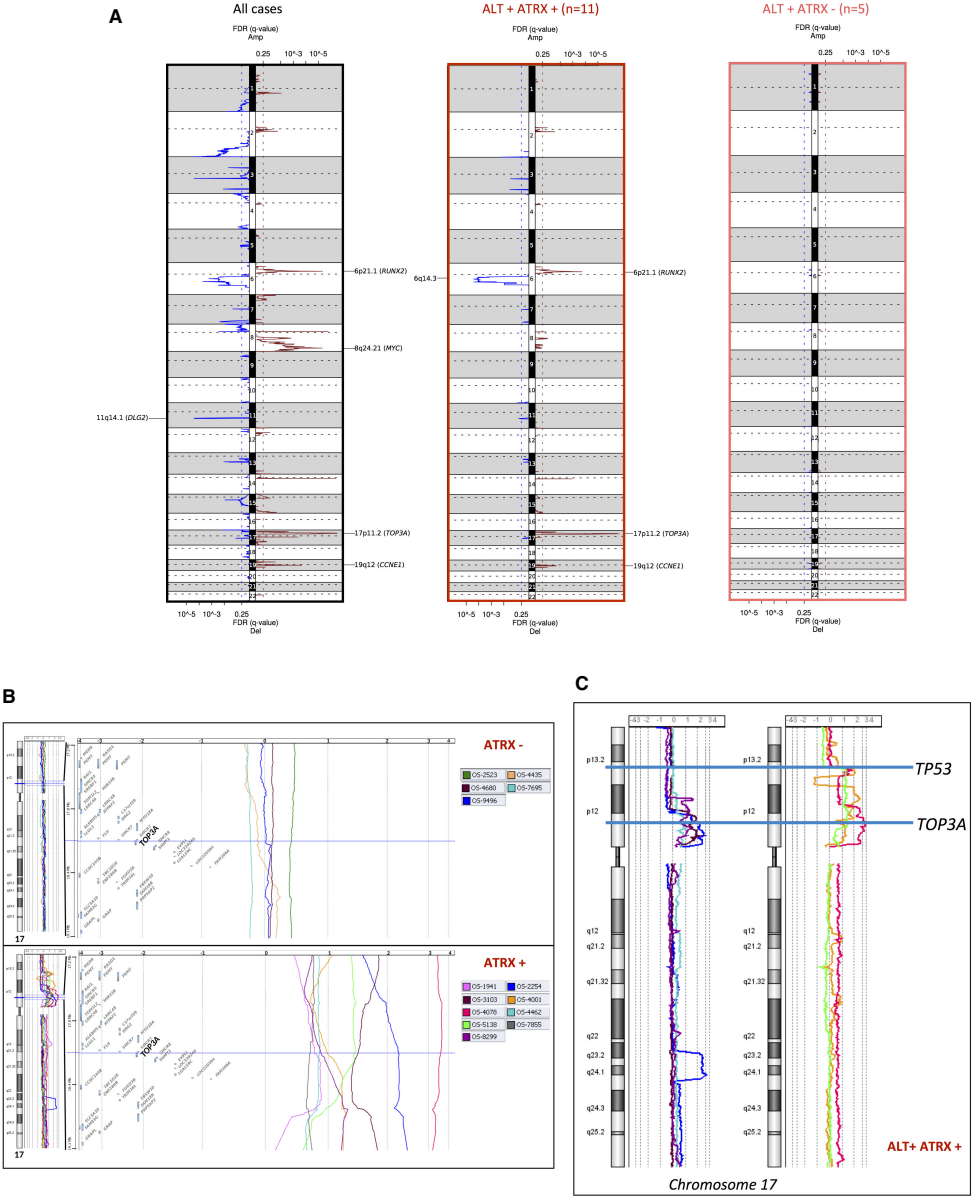
TOP3A amplification and α‐thalassemia/mental retardation syndrome X‐linked (ATRX) inactivation are mutually exclusive in alternative lengthening of telomeres (ALT) pediatric osteosarcoma Copy number alteration (CNA) profiles of osteosarcomas. Representation of the GISTIC analysis with false discovery rate (FDR (*q*‐value)) for all cases (*n* = 22; left panel), ALT‐positive/ATRX wild‐type (wt) cases (*n* = 11; middle panel), and ALT‐positive/ATRX‐mutated cases (*n* = 5; right panel).Gain/amplification of TOP3A gene region (17p11) is a genomic signature of ATRX‐wt osteosarcomas. For each ATRX‐mutated (top part) and ATRX‐wt (bottom part) case, from the left to the right, the chromosome 17 and regional genomic profiles were established, with CGH Analytics software. A focus was made within the genomic interval (17.25–19.11 Mb) of the short arm of chromosome 17 (hg38 human genome mapping; Build 38 from NCBI, December 2013 version) including TOP3. Color profiles corresponding to the different tumors are defined at the top of each group. Most ATRX‐wt cases show gain or amplification of this region (bottom part), whereas no ATRX‐mutated cases display this alteration (top part).Chromosome 17 copy number variation analysis showing amplification/gain of the *TOP3A* region that do not extend into the *TP53* gene in ALT ATRX‐wt tumors (left panel), and amplification of the *TOP3A* region for which *TP53* region is at the edge of the amplification/gain in ALT‐positive ATRX‐wt tumors (right panel).

### *TOP3A* amplification and *ATRX* mutation are exclusive genomic events in ALT‐positive high‐grade osteosarcoma

We next determined the genomic rearrangements of the 22 tumors. DNA copy number profiling using aCGH showed many regions of frequent gains and losses. Globally, ALT‐positive tumors appeared more rearranged than ALT‐negative tumors, but the difference was not significant, likely due to the low number of ALT‐negative tumors (Fig EV2B). Four regions (6p21.1, 8q24.21, 17p11.2, and 19q12) were amplified with high frequency in the tumors, while two were frequently deleted (6q14.3 and 11q14.1; Fig 3A and Table EV2).

We classified the most relevant genes that were either amplified (red bar) or deleted (blue bar) in the tumors and mainly considered the genes for which an amplification or a deletion occurred in at least three tumors (see Fig 2A). Within the gained regions, *ATAD2, MYC, RUNX2, RECQL4, ERBB2, PDGFRA*, and *CCNE1* were recurrently observed in the tumors and appear to be potential drivers, confirming previous studies in osteosarcoma (Chen *et al*, 2014; Kovac *et al*, 2015; Both *et al*, 2016; Bousquet *et al*, 2016; Sayles *et al*, 2019; Suehara *et al*, 2019; Guimarães *et al*, 2021).

Within the 17p11 amplified region, previously described to be frequently amplified in osteosarcomas (Forus *et al*, 1995; Tarkkanen *et al*, 1995; van Dartel *et al*, 2002, 2004; Bayani *et al*, 2003; Henriksen *et al*, 2003; Squire *et al*, 2003; Lau *et al*, 2004; Both *et al*, 2016), the *TOP3A* gene was always present in the amplicon, that is, all amplifications included *TOP3A*, among a number of other genes (Fig 3B). TOP3A caught our attention since it has an essential role in ALT (Tsai *et al*, 2006; Temime‐Smaali *et al*, 2008; Sobinoff *et al*, 2017; Pan *et al*, 2019; Loe *et al*, 2020; see below). Strikingly, *TOP3A* was amplified only in the ALT‐positive ATRX‐wt tumors and not in ALT‐positive ATRX‐mutated/deleted (ATRX‐mutated) tumors (71 vs. 0%; Fig 2A).

Interestingly, we found that in most tumors in which *TOP3A* is amplified (4,001, 4,078, 4,591, 4,665, and 5,136), the *TP53* gene was at the edge of the *TOP3A* amplification (Table EV3). This is particularly notable in tumors 4,001, 4,078, and 5,138, as opposed to tumors 2,254, 3,103, 4,464, and 8,299 where the copy number alteration (CNA) profile of the *TP53* region upstream *TOP3A* amplification is flat (Fig 3C and Table EV3). Although the *TP53* locus is represented on the array‐CGH chip by only two oligonucleotides, it seems likely that these amplifications result in breakage‐induced inactivation of at least one of the two alleles of TP53.

We also observed that *MMS21* (which cooperates with the SMC5/6 complex in DNA repair) and *NBS1* (from the MRE11/RAD50/NBS1 DNA repair complex), which both contribute to maintain telomere length by ALT (Compton *et al*, 2007; Potts & Yu, 2007; Zhong *et al*, 2007; Barroso‐González *et al*, 2019), were amplified with high frequency in the tumors (Fig 2A). However, in contrast to *TOP3A* and *NBS1*, *MMS21* amplification was also observed at high frequency in ALT‐negative tumors, suggesting that it does not have a specific role in the maintenance of ALT telomeres in pediatric osteosarcomas. Conversely, copy number losses of *SMARCAL1* and *XRCC5/KU80* were recurrently observed in ALT‐positive tumors, suggesting that depletion of these proteins may promote ALT (see Discussion).

Overall, these results suggest that amplification of *TOP3A* could be a new hallmark of the ALT‐positive/ATRX‐wt tumors.

### *TOP3A* is upregulated in ALT‐positive ATRX‐wt osteosarcomas

**Figure 4:**
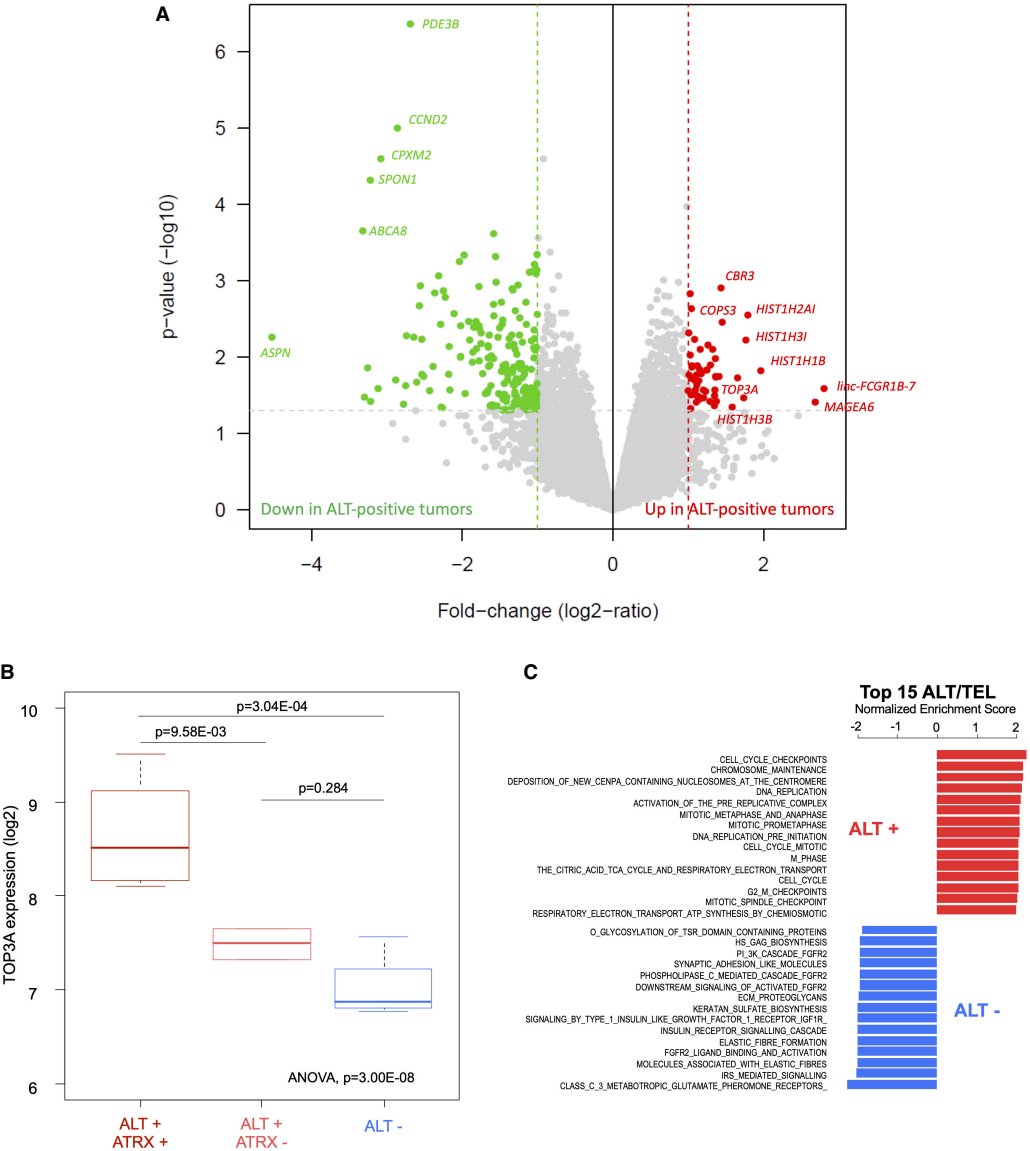
*TOP3A* is overexpressed in alternative lengthening of telomeres (ALT)‐positive/α‐thalassemia/mental retardation syndrome X‐linked wild‐type (ATRX‐wt) osteosarcomas Volcano plot of the supervised analysis of mRNA expression profiles of osteosarcomas according to ALT‐positive vs. ALT‐negative samples, showing upregulated (in red) and downregulated (in green) genes. In all, 12 tumors had sufficient RNA quality to be analyzed: seven ALT‐positive ATRX‐wt, two ALT‐positive/ATRX‐mutated and three ALT‐negative.TOP3A gene expression according to ALT and ATRX status: ALT^+^/ATRX‐wt (*n* = 7), ALT^+^/ATRX‐mut (*n* = 2) and ALT^−^ (*n* = 3). Box‐and‐whisker plot was defined with default parameters by median value (central band at the 50^th^ percentile), interquartile ranges (IQR, box limited by 25^th^ and 75^th^ percentiles) and whisker boundaries defined by minimum and maximum value. An ANOVA statistical test and Tukey's range test were used to compare modalities.Normalized enrichment score bar plot of top 15 significant REACTOME gene sets according to ALT‐positive vs. ALT‐negative GSEA analysis. Ontologies associated with ALT positivity are in red and those associated with ALT negativity are in blue.

**Figure EV4:**
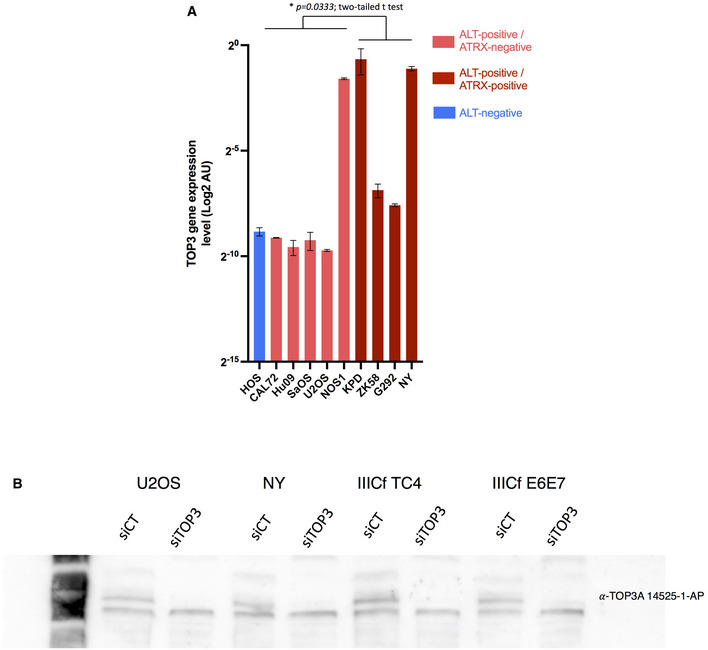
TOP3A expression in osteosarcoma cell lines TOP3A expression (by RT‐qPCR) in human osteosarcoma cell lines. Mean expression level of three technical replicates; error bars represent the mean ± SEM.TOP3A Western blots validating siRNA knockdowns in the indicated cell lines (U2OS, NY, IIIcf TC4, IIICf E6E7).

Next, we analyzed the transcriptomic profiles of the tumor samples (12 had good quality RNA) to highlight the differentially expressed genes (DEGs) between 9 ALT‐positive and 3 ALT‐negative tumors (Fig 4 and Table EV3). As shown in Fig 4A, the most upregulated genes in the ALT‐positive tumors were *CBR3, COPS3, MAGEA6*, and *TOP3A*, and a large number of histone genes from the *HIST1* cluster (*HIST1H2AI, HIST1H3I, HIST1H1B*, and *HIST1H3B*). Consistent with the amplification of the gene, *TOP3A* mRNA was overexpressed in the ALT‐positive tumors overall and in the ALT‐positive ATRX‐wt tumors but not in the ALT‐negative or ALT‐positive ATRX‐mutated tumors (Fig 4B), strengthening the point that *TOP3A* overexpression and *ATRX* mutation are exclusive genomic events in ALT‐positive osteosarcomas. The fact that several histone genes were overexpressed in ALT‐positive tumors compared to ALT‐negative tumors suggests that overexpression of histone genes may favor ALT in pediatric osteosarcomas (Fig EV4).

Globally, ontology analysis by Gene Set Enrichment Analysis (GSEA) in ALT‐positive versus ALT‐negative tumors revealed an enrichment in the expression of classical pathways related to chromosome duplication and transmission (Fig 4C and Table EV4). The prominence of DNA replication pathways in ALT‐positive tumors in the absence of any evidence of increased replication rate may reflect an increased need for restarting of stalled replication forks (Zhang & Zou, 2020). Conversely, ALT‐negative tumors were particularly enriched in genes related to the FGFR2‐associated signaling pathways that may be associated with telomerase reactivation in pediatric osteosarcoma (Greenfield *et al*, 2020).

### TOP3A expression in ALT‐positive ATRX‐wt and ALT‐positive ATRX‐mutated cell lines tumors as a validation of the tumor results

**Figure 5:**
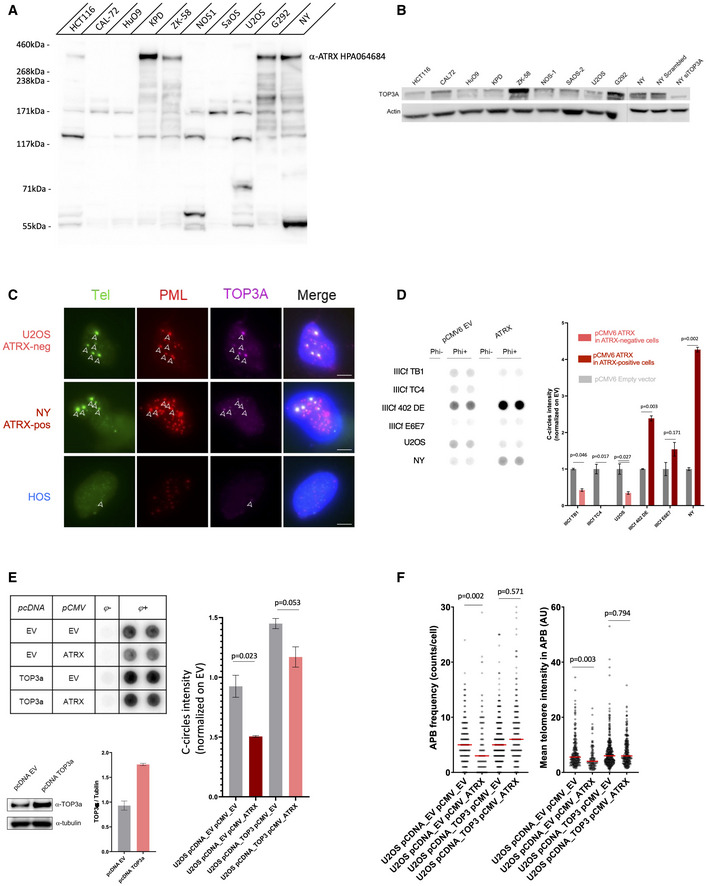
α‐thalassemia/mental retardation syndrome X‐linked (ATRX) is functional in alternative lengthening of telomeres (ALT)‐positive ATRX wild‐type (wt) cell lines and its ALT inhibitory function is counteracted by overexpression of TOP3A Western blot showing ATRX protein expression in nine osteosarcoma cell lines and HTC116 as positive control.Western blot showing TOP3A protein expression in nine osteosarcoma cell lines and NY siTOP3A as control.Telomere fluorescence *in‐situ* hybridization (FISH; green), and promyelocytic leukemia (PML; red) and TOP3A (purple) immunofluorescence (IF), showing TOP3A localization at APBs in both ATRX‐wt and ATRX‐mutated ALT‐positive cells. TOP3A foci are indicated by white arrows. Scale bars are 5 μm.C‐circle assay showing inverse effect of ATRX ectopic expression on C‐circle levels in ATRX‐mutated and ATRX‐wt cell lines. Error bars represent the mean ± SEM from *n* = 2 experiments, n.s. = non‐significant, *P < 0.05, Mann–Whitney test.C‐circle assay showing rescue of ATRX ectopic expression by TOP3A overexpression in U2OS osteosarcoma cells. Error bars represent the mean ± SEM from *n* = 2 experiments, n.s. = non‐significant, *P < 0.05, Mann–Whitney test. Western blot and quantification of TOP3A expression is shown for U2OS cells overexpressing TOP3A.ALT‐associated PML body (APB) frequency and mean telomeric DNA intensity in APBs according to TOP3A overexpression and ATRX transient expression; *n* = 150 cells scored per treatment, n.s., non‐significant, *P < 0.05; Mann–Whitney test.

**Figure EV5:**
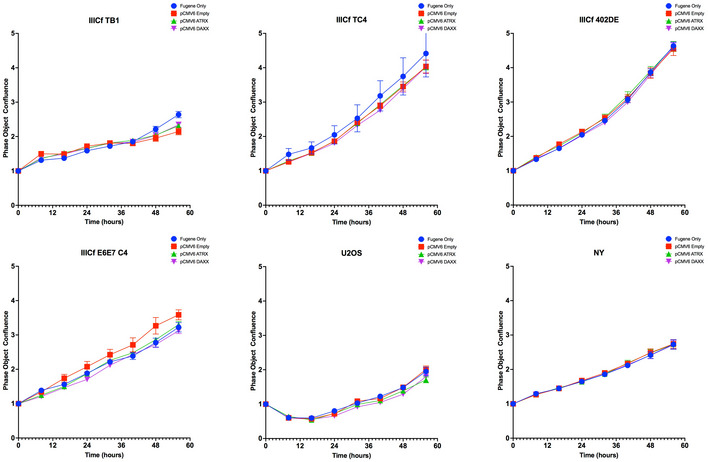
Effect of ectopic ATRX expression Growth curve (Points and error bars (±SEM) represent extrapolation of phase‐contrast object confluence assessed by Incucyte optical system from *n* = 3 technical replicates) according to transfection condition (FuGENE only, pCMV6‐empty, −ATRX, ‐DAXX).

One of the main discoveries of our study is that *TOP3A* amplification and *ATRX* mutation are mutually exclusive genomic events in ALT‐positive high‐grade osteosarcoma. However, in ALT‐positive/ATRX‐wt tumors, even if wt ATRX is normally expressed, the possibility exists that the ATRX protein might be non‐functional. We therefore sought to validate the results obtained from the tumors. We analyzed nine ALT‐positive human osteosarcoma‐derived cell lines (CAL72, HuO9, KPD, ZK58, NOS1, SaOS2, U2OS, G292, and NY; Lovejoy *et al*, 2012; Flynn *et al*, 2015). Sequencing data from the Sanger and Broad Institutes indicated that none of these cancer cell lines displayed a mutation in *ATRX* or *DAXX* (Tate *et al*, 2019), except NOS1 that had a homozygous loss of the *ATRX* genes (Loo *et al*, 2010). By Western blot, five out nine of these cell lines did not produce ATRX full‐length protein, while four cell lines (KPD, ZK‐58, G292, and NY) maintained ATRX protein expression (Fig 5A). We measured *TOP3A* mRNA expression in these nine osteosarcoma cell lines by quantitative reverse transcription polymerase chain reaction (RT‐qPCR) and found that its expression was higher in the ALT‐positive ATRX‐wt cell lines than in the ALT‐positive ATRX‐mutated cell lines, except NOS1 (*t* = 2.642, df = 7; *P* = 0.033) (Fig EV5). The NOS1 cell line turned out to have an elevated expression of *TOP3A* despite the fact it does not produce ATRX (see further). We monitored the expression of TOP3A by Western blot in these cell lines, which confirmed TOP3A overexpression in the ALT‐positive ATRX‐wt cell lines (Fig 5B).

By fluorescence *in‐situ* hybridization‐immunofluorescence (FISH‐IF), we found that TOP3A colocalized with telomeric DNA in APBs in both ATRX‐mutated and ATRX‐wt (ALT positive) cell lines (Fig 5C) indicating that the ATRX status did not modify the localization of TOP3A.

To determine whether the ATRX protein was functional or not in the ALT‐positive ATRX‐wt cell lines, we measured the effect of the ectopic expression of wt ATRX in ALT‐positive cell lines that were either ATRX‐wt or ATRX–mutated. We used the U2OS and NY cancer cell lines described above. In addition, we used immortalized IIICF cell lines that had been established from breast fibroblasts of a Li‐Fraumeni Syndrome patient (Maclean *et al*, 1994; Rogan *et al*, 1995; Bryan *et al*, 1997).

Because C‐circle levels are a robust readout of ALT and rapidly respond to perturbations in ALT activity (Henson *et al*, 2009), we determined whether C‐circle levels changed in response to transfection of a plasmid expressing wt ATRX in the U2OS, NY, and IIICF lines (Fig 5D). The level of C‐circles was reduced following ATRX expression in each of the ALT‐positive ATRX‐mutated cell lines examined (IIICF TB1, IIICF TC4, and U2OS) compared to empty vector‐transfected control cells. In contrast, in ALT‐positive ATRX‐wt cell lines transfected with wt ATRX (IIICF DE, IIICF E6E7, and NY), the C‐circle levels increased (Fig 5D). Of note, exogenous expression of ATRX did not affect the growth rate in any of the cell lines tested (Fig EV5). Therefore, while ATRX expression reduced C‐circles in ATRX‐mutated cells, it had rather the opposite effect on ATRX‐wt cells. These results suggest that wt ATRX is functional in the ALT‐positive ATRX‐wt cell lines.

To determine whether TOP3A overexpression is sufficient to overcome the ATRX‐dependent inhibition of ALT in U2OS expressing ectopic ATRX, we generated a U2OS cell line that stably overexpressed TOP3A (see Materials and Methods) and examined C‐circle levels (Fig 5E). As observed above, the level of C‐circles was reduced following pCMV‐ATRX transfection. However, the same cells also expressing TOP3A (pCMV‐TOP3A) exhibited only a mild reduction of the C‐circle level (Fig 5E). TOP3A overexpression restored APB frequency and mean telomere intensity in APB in U2OS transfected with pCMV‐ATRX (Fig 5F). Thus, TOP3A overexpression countered the ATRX‐mediated ALT inhibition, strengthening the results obtained with the clinical samples.

### *TOP3A* knockdown disrupts ALT phenotype and increases telomeric DNA damage

**Figure 6:**
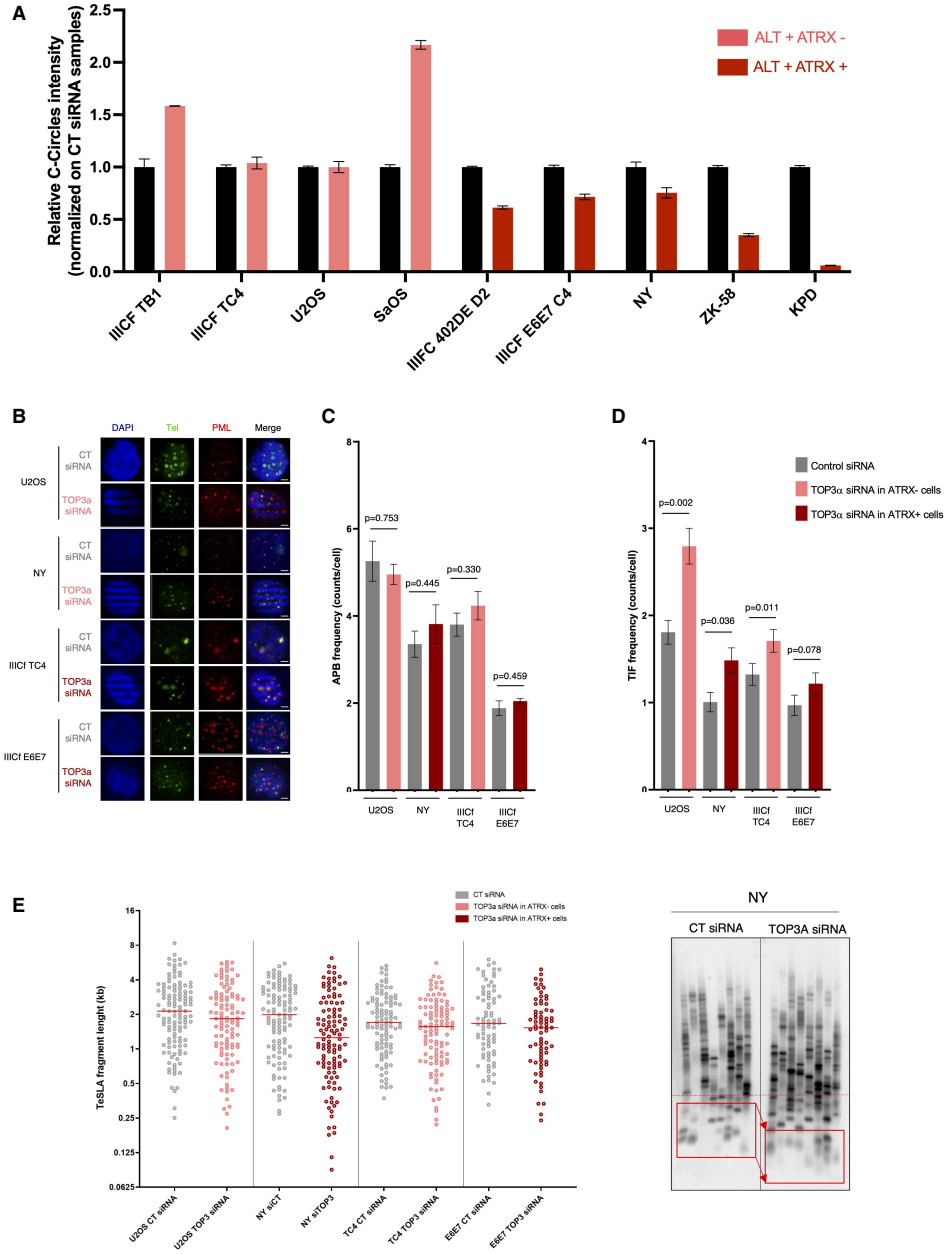
TOP3A inhibition affects alternative lengthening of telomeres (ALT) phenotype and leads to telomeric DNA damage Relative C‐circle intensity according to TOP3A KD in ALT‐positive ATRX‐mutated or ATRX wild‐type (wt) cell lines. Error bars represent the mean ± SEM from *n* = 2 experiments.Representative images of telomeric DNA (green) and promyelocytic leukemia (PML) protein (red) colocalizations (ALT‐associated PML body [APBs]) in osteosarcoma and *in vitro*‐immortalized ATRX‐mutated or ATRX‐wt cell lines according to TOP3A KD. Scale bars are 5 μm.Quantification of APB frequency in osteosarcoma and *in vitro*‐immortalized ATRX‐mutated or ATRX‐wt cell lines according to TOP3A KD; Error bars represent the mean ± SEM from *n* = 3 experiments, *n* = 150 cells scored per treatment, n.s., non‐significant; Mann–Whitney test.Effect of TOP3A KD on telomere dysfunction‐induced foci (TIFs) in osteosarcoma and *in vitro*‐immortalized cell lines; Error bars represent the mean ± SEM from *n* = 3 experiments, *n* = 150 cells scored per treatment, *P < 0.05, **P < 0.005; Mann–Whitney test.Distributions of telomere shortest length assay (TeSLA) fragments in osteosarcoma and *in vitro*‐immortalized ATRX‐mutated or ATRX‐wt cell lines according to TOP3A KD (left panel; each dot represents a TeSLA fragment), and representative TeSLA Southern Blot image for NY (right panel).

We next sought to determine the importance of TOP3A in ALT‐positive ATRX‐wt cells. To do this, we did a *TOP3A* knockdown (KD) in different ALT‐positive cell lines described above and analyzed its impact on ALT hallmarks (Fig EV4B). In the five ALT‐positive ATRX‐wt cell lines, *TOP3A* KD decreased C‐circles levels to different levels reflecting a disruption of the ALT phenotype. In contrast, *TOP3A* KD did not compromise C‐circle levels in ALT‐positive ATRX‐mutated cell lines (Fig 6A). Interestingly, *TOP3A* KD did not affect APB frequency (Fig 6B and C) in ALT‐positive cells, regardless of ATRX expression but induced an increase in telomeric damage‐induced foci (TIF) in U2OS, NY, and IIICF TC4 (Fig 6D). However, this increase in TIF numbers was associated with the appearance of very short telomeres (< 0.5 kb) only in the ALT‐positive ATRX‐wt cancer cell line NY (Fig 6E). Maintenance of telomeres in the ALT‐positive ATRX‐wt NY cell line was particularly sensitive to TOP3A depletion. Taken together, these results indicate that TOP3A overexpression contributes to maintenance of telomeres in ALT‐positive ATRX‐wt cells.

### TOP3A is required for proper BLM localization and promotes ALT DNA synthesis

**Figure 7:**
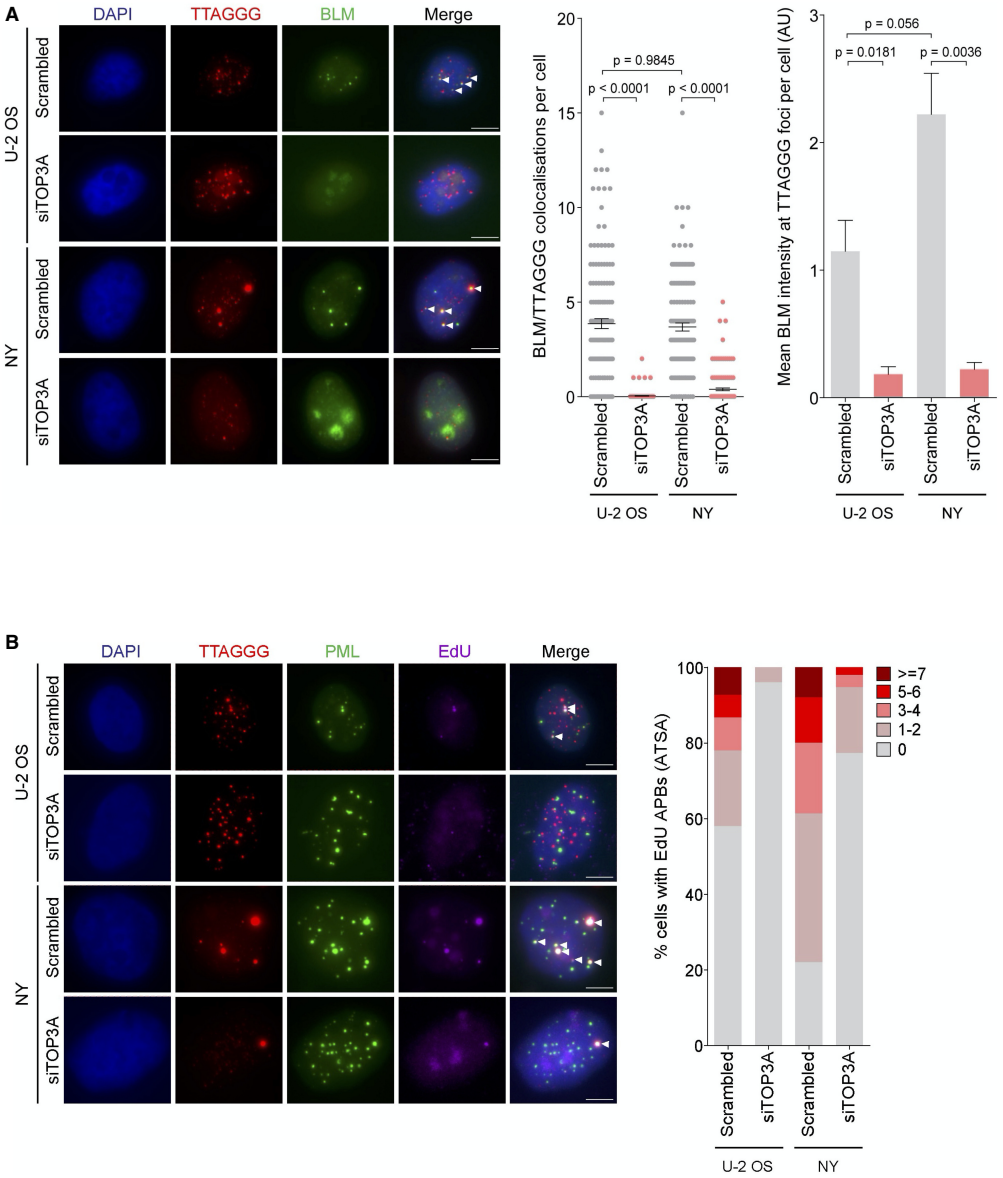
TOP3A depletion affects BLM localization at alternative lengthening of telomeres (ALT)‐associated PML body (APBs) and disrupts telomeric DNA synthesis Fluorescence *in‐situ* hybridization‐immunofluorescence (FISH‐IF) of telomeres (in red) and BLM (in green) in TOP3A‐depleted cells BLM foci at telomeres are indicated by white arrows. Error bars represent the mean ± SEM from *n* = 3 experiments, *n* = 150 cells scored per treatment, n.s. = non‐significant, **P < 0.01, Mann–Whitney test. Scale bars are 5 μm.
ATSA assay assessing ALT‐mediated telomeric DNA synthesis (telomere in red, EdU in purple) in promyelocytic leukemia (PML; in green) according to TOP3A KD. EdU signal at APB (telomere/PML) are indicated by white arrows. Approximately 150 cells were divided into five groups (0, 1–2, 3–4, 5–6*n*, and ≥ 7) based on the number of EdU + APBs. Scale bars are 5 μm.

Interestingly, our results indicate that TOP3A depletion particularly affects telomere maintenance in the ALT‐positive ATRX‐wt NY cell line. Because NY cells overexpress TOP3A, we hypothesized that this may lead to more BLM localization at telomeric sequences. Although not significant via Mann–Whitney, we observed an increase in the intensity of BLM at telomeres in the NY cells compared to U2OS cells (Fig 7A, right and left panels). We also found that TOP3A depletion clearly affects BLM localization at APBs in both cell lines (Fig 7A). Given this result, we measured ALT DNA synthesis (Sobinoff *et al*, 2017; Zhang *et al*, 2019) in the NY and UO2S cell lines (Fig 7B). We found that the NY cell line had a higher number of cells with EdU foci in APBs and that TOP3A depletion compromised ALT DNA synthesis in both cell lines (Fig 7B, right and left panels). Taken together, these results indicate that increased levels of TOP3A in NY cells correlate with increased levels of BLM at ALT telomeres and that TOP3A promotes ALT DNA synthesis. These results may explain the greater dependence of NY cells on TOP3A.

## Discussion

Although ALT is the TMM commonly utilized by osteosarcomas, many of these tumors have wt *ATRX*, a gene which is frequently mutated in ALT‐positive cancers (Ulaner *et al*, 2003; Sanders *et al*, 2004; Henson *et al*, 2005; Chen *et al*, 2014; Liau *et al*, 2015). The contrast with other types of sarcomas is illustrated by a study of ALT phenotype and ATRX status in 519 sarcoma samples which found loss of ATRX expression in approximately 50% of ALT‐positive leiomyosarcomas, undifferentiated pleomorphic sarcomas, and pleomorphic liposarcomas, whereas *ATRX* mutation and loss of expression was observed in only 30% of osteosarcomas (Liau *et al*, 2015). We show here that another mutational event is characteristic of *ATRX*‐wt osteosarcomas.

We found that ALT tumors carried either an *ATRX* mutation or an amplification of the 17p11.2 region as two mutually exclusive events. In this chromosomal region, we identified *TOP3A* amplification as the major genomic feature of the ALT tumors expressing wt ATRX. Gene expression analyses revealed that *TOP3A* was overexpressed in the ALT‐positive ATRX‐wt tumors compared to either ALT‐positive ATRX‐mutated or ALT‐negative tumors.

Amplification of the 17p11.2 region has been previously reported in osteosarcomas with a frequency ranging from 20 to 78% suggesting the presence of one or more oncogenes in this region (Forus *et al*, 1995; Tarkkanen *et al*, 1995; van Dartel *et al*, 2002, 2004; Bayani *et al*, 2003; Henriksen *et al*, 2003; Squire *et al*, 2003; Lau *et al*, 2004; Both *et al*, 2016). Of note, overexpression of genes belonging to the amplified region (*COPS3, PMP22, GID4, ARGHAP44, TOP3A, SHMT1*, and *RASD1*) was studied in osteosarcoma cell lines (Both *et al*, 2016). While TOP3A, SHMT1, and RASD1 overexpression resulted in increased proliferation, ARGHAP44, COPS3, and PMP22 overexpression had a stimulatory effect on migration and invasion of the cells. COPS3 and PMP22 overexpression additionally improved the ability of the cells to form new colonies (Both *et al*, 2016). However, the contribution of this region was not studied with respect to telomere maintenance previously. Here, we have shown that *TOP3A* amplification and overexpression is an alternative to ATRX inactivation, and the most frequent genetic event identified in ALT‐positive high‐grade pediatric osteosarcoma.

The hypothesis that TOP3A overexpression was instrumental in maintaining ALT in ATRX‐wt cells was confirmed by *in vitro* experiments showing first that TOP3A overexpression could restore ALT features in ALT‐positive ATRX‐mutated cell lines transfected with wt ATRX and second that *TOP3A* KD disrupted the ALT phenotype with a concomitant increase in telomeric DNA damage in ALT‐positive ATRX‐wt cells. TOP3A is a type I DNA topoisomerase operating within the BLM‐TOP3A‐RIM1 complex (known as the BTR complex) that suppresses crossing‐over during HR (Wu & Hickson, 2003). TOP3A was previously shown to be localized at ALT telomeres and to be required to maintain ALT telomeres (Tsai *et al*, 2006; Temime‐Smaali *et al*, 2008). TOP3A was further shown to promote POLD3‐dependent telomere synthesis through a mechanism analogous to break‐induced replication (Dilley *et al*, 2016; Sobinoff *et al*, 2017; Lu *et al*, 2019). The central role of the BTR complex in ALT was further underscored by the recent discovery that its localization at telomere ends is essential for ALT activity (Loe *et al*, 2020; Zhang *et al*, 2021). Here we have shown that TOP3A depletion affects BLM localization at APBs and compromises ALT DNA synthesis in U2OS and NY cell lines that are ATRX mutated and ATRX‐wt, respectively. However, we found that the NY cell line has more BLM at APBs and higher levels of ALT DNA synthesis consistent with the fact that NY cells overexpress TOP3A and are more sensitive to TOP3A depletion. TOP3A has also been described to have a role in the processing of ultra‐fine bridges that connect the separating sister DNA molecules during anaphase at specific unreplicated genomic loci, including telomeres (Sarlós *et al*, 2018), suggesting that TOP3A plays a role in mitotic DNA synthesis (MiDAS; Özer & Hickson, 2018). Moreover, recent results indicated that reconstituted PML bodies consisting of polySUMO/polySIM condensates targeting telomeres were able to cluster telomeres and generate ALT telomeres through a process mediated by BLM and RAD52 (Min *et al*, 2019; Zhang *et al*, 2019, 2020).

Collectively, these studies reveal that BIR, MiDAS, and ALT share molecular processes in which TOP3A is a major actor (Epum & Haber, 2021). We speculate that in tumors undergoing replication stress, TOP3A overexpression promotes these molecular processes, accounting for its overexpression being a critical contributor to ALT activity in tumors.

Strikingly, we observed that amplification of the 17p11.2 region may both amplify TOP3A and disrupt at least one allele of *TP53* in most ATRX‐wt tumors. Analysis of the TP53 status in the tumors carrying an amplified 17p11.2 region raises the interesting hypothesis that the 17p11 amplification could do “double duty,” on the one hand amplifying *TOP3A* and on the other hand inactivating *TP53*.

Beside *TOP3A* amplification, our study revealed alterations in other genes that have been implicated in ALT. *NBS1*, the depletion of which results in inhibition of ALT in human cell lines (Compton *et al*, 2007; Zhong *et al*, 2007), was amplified in ALT‐positive tumors. *SP100*, which sequesters the MRN complex thereby suppressing ALT (Jiang *et al*, 2005), had a loss of copy number. These results highlight the importance of the MRN complex in the formation of ALT telomeres in these pediatric tumors. Loss of *KU86* may also play a role in ALT, as suggested by several studies (Zellinger *et al*, 2007; Wang *et al*, 2009; Yu *et al*, 2015). Of note, we did not find alterations in SLX4IP that was reported to antagonize BLM activity during ALT Maintenance (Panier *et al*, 2019).

We found mutations in genes that organize the structure of chromatin. Although not previously reported in osteosarcomas, mutations in *KMT2B/MLL2*, *KMT2C/MLL3*, or *KMT2D/MLL4* that catalyze mono‐ or di‐methylation of H3K4 (Hyun *et al*, 2017) are frequently observed in many types of cancer (Rao & Dou, 2015; Fagan & Dingwall, 2019). Interestingly, it was recently reported in mouse embryonic stem cells deleted for *ATRX*, *TP53*, and *TERT* that inhibiting KDM4B/JMJD2B (which demethylates H3K9 and H3K36) drives ALT activation (Udugama *et al*, 2021). Consistent with this observation, we found that among the five ALT‐positive ATRX‐mutated tumors, two carried mutations in *TP53* and *KDM4A/JMJD2A* (E23K and K463Q; that also demethylates H3K9 and H3K36), suggesting that simultaneous inactivation of *TP53*, *ATRX*, and *KDM4A/JMJD2A* may also drive ALT in pediatric osteosarcoma.

In pediatric high‐grade glioma, the G34R mutation in H3.3 triggers ALT, irrespective of the ATRX status. In contrast, only a subset of H3.3‐K27M tumors activate ALT (Minasi *et al*, 2021). This result is to be compared with a recurrent amplification of 17p11.2 targeting TOP3A and mutually exclusive with ATRX deletion/mutations that has been identified in diffuse intrinsic pontine glioma with a H3.3‐K27M mutation (Mackay *et al*, 2017). Together with our data, these results suggest that under conditions that reprogram the epigenetic landscape, ATRX inactivation or TOP3A amplification may represent two independent ways of promoting ALT.

We found that of the 13 tumors displaying mutations in histones genes of the *HIST1* cluster, 11 were ALT positive irrespective of their *ATRX* status. Two mutations (in *HIST1H3A* and *HIST1H4H*) either introduced a frameshift or modified the C‐terminal region known to be crucial for the interaction with the histone chaperone ASF1 (Natsume *et al*, 2007), the depletion of which leads to the rapid induction of ALT in primary human lung fibroblasts (O'Sullivan *et al*, 2014). In addition, we observed that many histone genes of the *HIST1* cluster were upregulated in ALT‐positive tumors compared to ALT‐negative tumors. We favor the idea that overexpression of canonical histone genes in the ALT‐positive tumors might create an imbalance between canonical histones and histone variants (Maya Miles *et al*, 2018), which may generate replication stress promoting ALT.

Finally, the analysis of clinical data revealed that, among patients with good response to neoadjuvant chemotherapy, ALT positivity of the pre‐treatment biopsy was associated with worse DFS than ALT negativity (4/9 events vs. 0/4 at 126.2 of median follow‐up, respectively). This observation, which needs to be validated in a larger cohort, might suggest the relevance of studying TMM as a biomarker in high‐grade pediatric osteosarcomas to reassign ALT‐positive good responders as candidates for alternative adjuvant therapy after surgery. To date, there is no prognostic marker able to differentiate good responders from poor responders at diagnosis. This result is consistent with many other studies showing that, with the notable exception of glioblastomas, ALT positivity is associated with unfavorable clinical outcome of most tumor types (Henson & Reddel, 2010).

In conclusion, our study identified a number of genomic features associated with ALT, providing new targetable proteins and potential therapeutic opportunities for osteosarcoma. The results emphasize the potential interest in developing TOP3A inhibitors for treatment of ALT‐positive *ATRX*‐wt osteosarcomas.

## Materials and Methods

### Patient cohorts and tumor samples

Frozen tumor samples of 22 high‐grade osteosarcomas from pediatric patients treated at Timone Hospital (Marseille, France) were retrospectively obtained. At the time of these diagnostic biopsies, all patients had strictly localized diseases and were naïve of any pre‐treatment. Diagnosis of osteosarcoma was made by an expert pathologist of the French Sarcoma Group and all specimens were conventional high‐grade osteosarcomas. Normal tissue was obtained from surgical resection and after neo‐adjuvant chemotherapy for 21 patients. All patients or their legal guardian provided written informed consent (also see Ethics Declaration below).

### Ethics declaration

This study was approved by the Aix‐Marseille University Research Ethics Committee. Tumor material was obtained from the Timone Hospital biobank (Tumorothèque et Banque de Muscles, Hôpital de la Timone—Registration no. AC‐2018‐3105 at the Ministry of Higher Education, Research and Innovation). Signed and written formal consent was obtained from all patients or their legal guardian. The experiments conformed to the principles set out in the WMA Declaration of Helsinki and the Department of Health and Human Services Belmont Report.

### C‐Circle assay

C‐circles were amplified with Phi29 polymerase using dATP, dTTP, and dGTP overnight. Products were dot blotted onto Biodyne B membranes (Pall) and pre‐hybridized in PerfectHyb Plus (Sigma) for at least 30 min. γ‐[^32^P]‐ATP‐labeled telomeric C‐probe (CCCTAA)_4_ was then added and blots were hybridized overnight at 37°C (Henson *et al*, 2009). Blots were washed with 0.5× SSC, 0.1% SDS three times for 5 min each, then exposed to a PhosphorImager screen. Imaging was performed on the Typhoon FLA 7000 system (GE Healthcare) with a PMT of 750 V.

### DNA and RNA extractions

DNA and RNA extraction from tumor frozen samples was performed using the All prep DNA/RNA kit from QIAGEN. Frozen biopsies were first ground in a mortar containing liquid nitrogen to obtain a fine powder. Then, we proceeded according to the manufacturer's instructions. DNA amounts were quantified with Nanodrop® and QUBIT® (Qubit DNA HS assay kit). RNA quality was controlled on an Agilent Bioanalyzer (Agilent Technologies, Massy, France) assessing RNA integrity Number (RIN) for each sample.

To extract DNA from cell lines, cells were harvested via trypsinization, washed in PBS, and resuspended in lysis buffer (50 mM Tris–HCl, 100 mM NaCl, 50 mM ethylenediamine tetraacetic acid (EDTA), 0.5% SDS, pH 8). Lysed cells were subjected to RNase A (50 μg/ml) treatment for 20 min at room temperature, followed by protein digestion with 400 μg/ml proteinase K (Invitrogen) overnight at 55°C. DNA was extracted using three rounds of phenol/chloroform extraction followed by ethanol precipitation. Total RNA was isolated using the RNeasy mini kit (Qiagen) and DNase‐treated to remove genomic DNA.

### Library, sequencing, and bioinformatics analysis

tNGS was applied to a custom‐made panel of 755 “cancer‐associated” and “actionable” genes (Table EV1). For each of the 22 clinical samples, the DNA libraries of all coding exons and intron–exon boundaries of all genes were prepared using the HaloPlex Target‐Enrichment‐System (Agilent, Santa Clara, CA, USA), as previously described (Collette *et al*, 2015). Sequencing was done using the 2 × 150‐bp paired‐end technology on the Illumina NextSeq500 platform, according to the manufacturer's instructions (Illumina, San Diego, CA, USA). Sequence data were aligned to the human reference genome (UCSC hg19) and analyzed, as previously described (Collette *et al*, 2015; Bertucci *et al*, 2016). Computing resources for this study were provided by the computing facilities DISC (Datacenter IT and Scientific Computing) of the Centre de Recherche en Cancérologie de Marseille.

### DNA copy number profiling

For each sample, the genomic profile was established by using aCGH onto high‐resolution 4 × 180 K CGH‐microarrays (SurePrint G3‐Human CGH‐Microarray, Agilent Technologies). Human female DNA was used as reference (G152A, Promega, Madison, WI, USA). Both experimental and analytic methods have been previously described (Adélaïde *et al*, 2007). All probes for aCGH were mapped according to the Genome Reference Consortium Human Build 37 (CGCh37/Hg19; https://www.ncbi.nlm.nih.gov/assembly/GCF_000001405.13/). We used two different threshold values (log_2_ ratio > ¦0.2¦ and ¦0.5¦) to distinguish low‐ (gain/loss) from high‐(amplification/deletion) level CNA, respectively. Percentage of altered genome was the number of probes above the threshold divided by the total number of probes for autosomal chromosomes. To identify the overall altered regions, we used the GISTIC2.0 algorithm with alteration thresholds set to 0.15 and with a corrected threshold probability (*q* < 0.25) to define a statistically relevant region.

### Transcriptomic analyses

DNA microarrays were used to define the transcriptional profiles of 12 tumor samples with a RIN ranging from 8.1 to 9.9. Experiments were done as recommended by the manufacturer (Affymetrix, Thermo Fisher) from 100 ng of total RNA for each sample using the GeneChip™ WT PLUS Reagent Kit and the Affymetrix GeneChip™ HuGene 2.0 ST arrays. Expression data were normalized by RMA with the nonparametric quantile algorithm in R using Bioconductor and associated packages (version 3.5.2; http://www.cran.r‐project.org/). Supervised analysis comparing the expression profiles between sample classes was done using a moderated *t*‐test with empirical Bayes statistic^100^ included in the limma R package (version 3.38.3). Significantly, DEGs were defined by the following thresholds: *P*‐value < 0.05 and fold change FC > ¦2x¦. Ontology analysis was done by the GSEA algorithm with the Reactome pathway database included in the MSigDB (version 7.2) ^101^. GSEA was done with 1000 gene‐set permutations as parameters and significantly DEG sets were defined by the following thresholds: *P*‐value < 0.05 and *q*‐value < 0.25.

### Immunochemistry

Immunohistochemistry was performed using ATRX (1:500, Sigma Aldrich) and DAXX (1500, Sigma Aldrich) antibodies. The immunohistochemical protocol included deparaffinization, hydration, antigen retrieval, primary antibody incubation, and detection and visualization as per the manufacturer's instructions.

### Telomere shortest length assay

The TeSLA method was performed as described (Lai *et al*, 2017). Briefly, 50 ng of genomic DNA was added to a final volume of 20 μl ligation buffer containing 1,000 units of T4 DNA ligase (New England Biolabs), 1× Cut Smart Buffer (New England Biolabs), 1 mM ATP, and 1 nM of TeSLA telorettes (TeSLA Telo 1–6) and incubated at 35°C for 16 h followed by heat inactivation at 65°C for 10 min. After ligation, genomic DNA was digested using a set of restriction enzymes (2 U each of CviAII, BfaI, NdeI, and MseI, New England Biolabs) and then treated with 1 U of Shrimp Alkaline Phosphatase (rSAP, New England Biolabs) at 37°C for 60 min in a final volume of 50 μl. This mixture was subsequently heat inactivated at 80°C for 20 min and 10 μl of sample was added to 10 μl of adapter ligation mix (1 μM AT adapter, 1 μM TA adapter, 1 mM ATP, 1× Cut Smart Buffer and 2,000 units of T4 DNA Ligase) and incubated at 16°C for 16 h. After adapter ligation, the sample was heat inactivated at 65°C for 10 min and subsequently diluted to a concentration of 15 pg DNA/μl (1:25 dilution). For each sample analyzed, we performed eight independent PCRs (94°C for 2 min followed by 26 cycles of 94°C for 15 s, 60°C for 30 s, and 72°C for 15 min) using a total of 25 μl mix containing 30 pg DNA, 2.5 U FailSafe enzyme (Epicenter), 1× FailSafe buffer H (Epicenter), and 250 nM primers (adapter and TeSLA TP). PCR products were electrophoresed on a 0.85% agarose gel (1.5 V/cm for 19 h). The gels were dried for 75 min at 60°C, denatured in 0.5 M NaOH/1.5 M NaCl for 1 h, and neutralized in 0.5 M Tris–HCl (pH 8.0)/1.5 M NaCl for 1 h. Gels were then rinsed in 2× SSC and prehybridized in Church buffer (250 mM sodium phosphate buffer, pH 7.2, 7% [wt/vol] SDS, 1% [wt/vol] BSA fraction V grade, and 1 mM EDTA) for 2 h at 37°C. Finally, gels were hybridized overnight with a γ‐[^32^P]‐ATP‐labeled (TTAGGG)_3_ oligonucleotide probe, washed three times in 0.1× SSC for 15 min at 37°C, and exposed to a PhosphorImager screen overnight. Images were analyzed using MATLAB‐based software to detect and annotate the size of telomere bands including the percentage of shortest telomeres and average telomere lengths.

### Cell culture and cell lines

All cell lines were cultured in Dulbecco's modified Eagle's medium supplemented with 10% (v/v) fetal bovine serum in a humidified incubator at 37°C with 5% CO_2_. HCT116, CAL72, HuO9, KPD, ZK58, NOS1, SaOS2, U2OS, G292, and NY cell lines were obtained from the American Type Culture Collection. IIICF cell lines are *in vitro*‐immortalized breast fibroblast cells that were established from an individual with Li–Fraumeni syndrome (Maclean *et al*, 1994; Rogan *et al*, 1995; Bryan *et al*, 1997). Cell lines were authenticated by 16‐locus short‐tandem‐repeat profiling and tested for mycoplasma contamination by CellBank Australia (Children's Medical Research Institute, Westmead, NSW, Australia).

### Transfection

An ATRX expression vector, pCMV6‐Entry‐ATRX, and the empty vector pCMV6‐Entry were obtained from OriGene. Transfection with each vector was performed using FuGENE reagent (Promega). Cells were harvested at 72 h post‐transfection and analyzed as described. Stable TOP3A‐overexpressing U2OS cell lines were generated as follows: cells were seeded at 2 × 10^5^ per 6‐well plates and reverse transfected with 1 μg DNA of either pcDNA‐TOP3A or pcDNA‐Empty plasmid (courtesy of JF Riou), using FuGENE‐6 reagent (Promega) according to the manufacturer's instructions. G418 (400 μg/ml) was added after 24 h for selection over 1 month. Cells were continuously cultured in G418 to ensure overexpression. Protein overexpression was confirmed via Western blot analysis.

### Western blot

Cells were collected and lysed in radioimmunoprecipitation assay buffer. Proteins were resolved by electrophoresis using a Tris‐acetate gel or 4–12% Bis‐Tris gel (Invitrogen). Transferred membranes were blocked in 5% milk and incubated with primary antibody overnight. Antibodies were used against ATRX (Sigma HPA064684, SC10078, and SC1540; 1:800 dilution), DAXX (Sigma HPA008736; 1:800 dilution), Vinculin (T5191; 1:600 dilution), γ‐tubulin (Sigma, T5192; 1:1,000 dilution), and TOP3A (proteintech 14,525‐1‐AP; 1:600 dilution).

### RNA interference

Two TOP3A Silencer Select siRNAs were designed and synthesized by Life Technologies (s14311 & s224746), and Silencer Select RNAi siRNA Negative Control #2 (#4390847) was used as the control siRNA. Cell suspensions were transfected at 20–50% confluency with Lipofectamine RNAiMAX (Life Technologies) at a final siRNA concentration of 30 nM. Culture medium was changed after 48 h and cells were harvested for analysis 72 h post‐transfection. KD efficiency was validated by western blot analysis.

### Quantitative reverse transcription PCR

KD experiments using siRNA were verified by qRT–PCR. Total RNA was isolated using the RNeasy mini kit (Qiagen) and DNase‐treated to remove genomic DNA. Reverse transcription was performed with 2 μg isolated RNA, 500 ng oligo(dT)15 primer, 40 U RNasin, 0.5 mM dNTPs, and 20 U M‐MLV‐Reverse Transcriptase (Promega). Quantitative PCR was performed using SYBR Green qPCR Master Mix (Roche) according to the manufacturer's instructions. Primer sequences are supplied as supplementary data (Table EV4). PCRs were performed on cDNA equivalent to 100 ng total RNA and carried out for 40 amplification cycles, followed by melt curve analysis. For each sample, a replicate omitting the reverse transcription step was included as a negative control. Primer PCR efficiencies were calculated by standard curve. Real‐time data were analyzed using the ΔΔC(*t*) method, with *GAPDH* as the reference gene, and expressed as fold‐change relative to the appropriate control (±SEM).

### Immunofluorescence and fluorescence *in‐situ* hybridization

Cells were grown on cover slips. Slides were subjected to pre‐extraction by incubation in KCM permeabilization solution (120 mM KCl, 20 mM NaCl, 10 mM Tris, 0.1% (v/v) Triton X‐100) for 10 min. Slides were then washed in PBS, fixed at room temperature for 10 min in PBS with 4% (v/v) formaldehyde. For tumor samples, 4 μm paraffin sections were cut and dewaxed. Slides were rehydrated then microwave heated to 120°C in 90% glycerol, 10 mmol/l Tris (pH 10.5), 1 mmol/l EDTA, and maintained at 110–120°C for 15 min. The slides were cooled and rinsed in PBS. All subsequent treatments of cell lines and paraffin sections were identical. Cells were blocked with 100 μg/ml DNase‐free RNase A (Sigma) in antibody‐dilution buffer (20 mM Tris–HCl, pH 7.5, 2% (w/v) BSA, 0.2% (v/v) fish gelatin, 150 mM NaCl, 0.1% (v/v) Triton X‐100, and 0.1% (w/v) sodium azide) for 1 h at room temperature. Antibodies were used against PML (Santa Cruz, sc‐966; Goat; 1:400 dilution), γ‐H2AX (Merck Millipore, Mouse, 1:500 dilution), BLM (Bethyl Laboratories, A300‐110A; Rabbit; 1:500 dilution), and TOP3A (Temime‐Smaali *et al*, 2008; Rabbit; 1:1,000 dilution). Slides were incubated with primary antibody diluted in antibody‐dilution buffer for 1 h at room temperature, washed in phosphate‐buffered saline‐Tween‐20, and incubated with appropriate AlexaFluor secondary antibodies diluted in antibody‐dilution buffer for 1 h at room temperature in a humidified chamber. Subsequent telomere FISH was performed. Slides were washed in PBST and fixed for 10 min in PBS with 4% (v/v) formaldehyde at room temperature. Slides were then subjected to a graded ethanol series (70% (v/v) for 3 min, 90% (v/v) for 2 min, and 100% for 2 min) and allowed to air‐dry in the dark. Dehydrated slides were then overlaid with 0.3 μg/ml TAMRA–OO‐(CCCTAA)_3_ or Alexa 488‐OO‐(CCCTAA)3 telomeric PNA probe (Panagene) in PNA hybridization solution (70% (v/v) deionized formamide, 0.25% (v/v) NEN blocking reagent (PerkinElmer), 10 mM Tris–HCl, pH 7.5, 4 mM Na2HPO4, 0.5 mM citric acid, and 1.25 mM MgCl2), denatured at 80°C for 3 min, and hybridized at room temperature overnight. Slides were washed in PNA wash A and then in PNA wash B for 15 min each. 4',6‐diamidino‐2‐phénylindole (DAPI) was added at 50 ng/ml to the final wash. Finally, slides were rinsed briefly in deionized water and mounted in DABCO anti‐fade mounting media.

### ATSA assay (ALT telomere DNA synthesis in APBs)

To visualize DNA synthesis at telomeres, cells were treated with 10 μM of RO‐3306 for 18 h for G2 synchronization. G2 synchronized cells were then pulsed with 10 μM EdU for 1 h. Cells were permeabilized in KCM permeabilization solution (120 mM KCl, 20 mM NaCl, 10 mM Tris, 0.1% (v/v) Triton X‐100) for 10 min, then fixed with 4% formaldehyde PBS solution for 10 min. The Click‐iT® Alexa Fluor 647 azide reaction (Invitrogen) was then performed according to the manufacturer's instructions. Indirect immunofluorescence for PML and telomere‐FISH were then performed according to the relevant methods section.

### Automated image analysis

ZEN microscopy images (.czi) were processed into extended projections of z‐stacks using ZEN desk 2011 software (Zeiss) and imported into Cellprofiler v2.1.1 (29) for analysis. The DAPI channel was used to mask individual nuclei as primary objects. Foci within each segmented nucleus were identified using an intensity threshold‐based mask. Any given object was considered to be overlapping another object when at least 80% of the first object's area was enclosed within the area of a second object.

## Author contributions

**Alexandre de Nonneville:** Conceptualization; formal analysis; validation; supervision; investigation; visualization; methodology; writing – original draft. **Sébastien Salas:** Resources; project administration. **François Bertucci:** Investigation; methodology; writing – review and editing. **Alexander P Sobinoff:** Investigation; methodology. **José Adélaïde:** Investigation; methodology. **Arnaud Guille:** Investigation. **Pascal Finetti:** Investigation; methodology. **Jane R Noble:** Project administration. **Dimitri Churikov:** Conceptualization; supervision; investigation; methodology. **Max Chaffanet:** Methodology. **Elise Lavit:** Investigation. **Hilda A Pickett:** Investigation; methodology. **Corinne Bouvier:** Resources; investigation; methodology. **Daniel Birnbaum:** Conceptualization; supervision; funding acquisition; validation; investigation; writing – review and editing. **Roger R Reddel:** Conceptualization; formal analysis; funding acquisition; validation; investigation; methodology; writing – review and editing. **Vincent Géli:** Conceptualization; formal analysis; supervision; funding acquisition; validation; investigation; writing – original draft; project administration.

## Disclosure and competing interests statement

The authors declare that they have no conflict of interest.

**The paper explained**

**Problem**

To maintain their telomeres, most human cancer cells upregulate telomerase, while the rest uses a mechanism based on HR‐mediated DNA replication ALT. High‐grade osteosarcomas are highly aggressive bone tumors that mainly occur in children and adolescents, with limited treatment options. Strikingly, osteosarcomas exhibit a high frequency of ALT. In contrast to many other tumors, ATRX, a chromatin remodeler, is mutated in only 30% of ALT‐positive osteosarcomas, indicating that these may not be the only alterations responsible for ALT.

**Results**

We characterized non‐metastatic, non‐pre‐treated, high‐grade pediatric osteosarcomas and uncovered that more than 70% of these tumors maintained their telomeres via the ALT pathway. In the ALT tumors, TOP3A gene amplification and overexpression was more prevalent than ATRX loss‐of‐function. Because ATRX mutation and TOP3A overexpression were mutually exclusive, overexpression of TOP3A appeared as an alternative mechanism driving ALT in pediatric osteosarcoma. In ALT‐positive human osteosarcoma‐derived cell lines, TOP3 overexpression also correlated with the presence of ATRX. Moreover, TOP3A was required to localize the BLM helicase to telomeres and promote ALT‐associated DNA synthesis in ALT osteosarcoma cell lines. Several genes whose alteration is associated with ALT in pediatric osteosarcoma were additionally identified, revealing new mechanistic insights into telomere maintenance in these tumors. Overall, these results identify TOP3A or TOP3A downstream signaling as a potential therapeutic target.

**Impact**

Amplification of TOP3A is a new hallmark of the ALT mechanism in tumors that are otherwise wt for ATRX. The discovery that TOP3A amplification/overexpression is mutually exclusive with ATRX mutation constitutes a strong argument for the involvement of TOP3A in oncogenesis. Our work therefore positions TOP3A action at telomeres as a prognostic marker and a new potential therapeutic target in ALT‐associated pediatric osteosarcomas.

## Supporting information



Expanded View Figures PDF

Table EV1

Table EV2

Table EV3

Table EV4

PDF+

## Data Availability

The datasets and computer code produced in this study are available at the ArrayExpress database with the following accession number: RNA Affymetrix GeneChip: E‐MTAB‐11743 (Title: Alternative Lengthening of Telomeres (ALT) and ATRX inactivation in high‐grade pediatric osteosarcomas. Permanent link: https://www.ebi.ac.uk/arrayexpress/experiments/E‐MTAB‐11743); CGH: E‐MTAB‐11747 (Title: Genome profiling by comparative genomic hybridization (CGH) in pediatric osteosarcoma. Permanent link: https://www.ebi.ac.uk/arrayexpress/experiments/E‐MTAB‐11747).
