# Supplementary material for: TOP3A amplification and ATRX inactivation are mutually exclusive events in pediatric osteosarcomas using ALT
Source: EMBO Mol Med. 2022 Aug 3;14(10):e15859. doi: 10.15252/emmm.202215859 (PMC9549729; doi:10.15252/emmm.202215859)
Supplement: Supplementary file 1 — Expanded View Figures PDF [file EMMM-14-e15859-s006.pdf]

Expanded View Figures

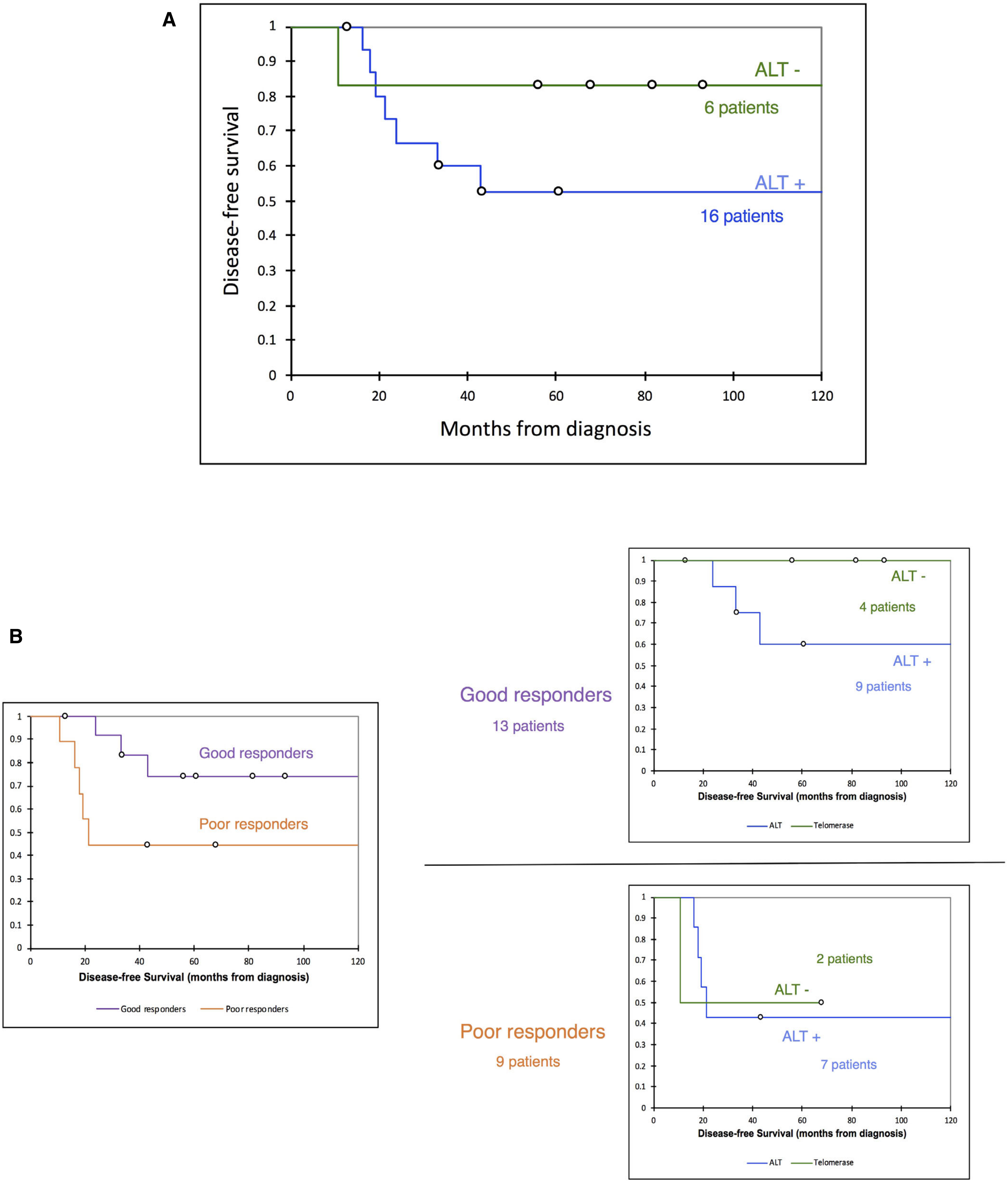

Figure EV1.

◀ **Figure EV1. Survival analysis according to ALT.**

A Disease-free survival curves of patients with ALT-positive and ALT-negative tumors.

B Disease-free survival curves of good and poor responders to neoadjuvant chemotherapy (left panel), then dichotomized according to ALT status of tumors (right panel).

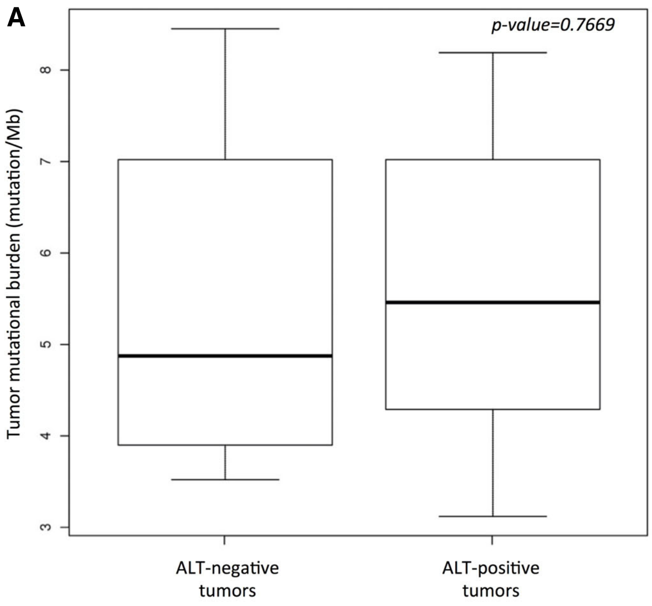

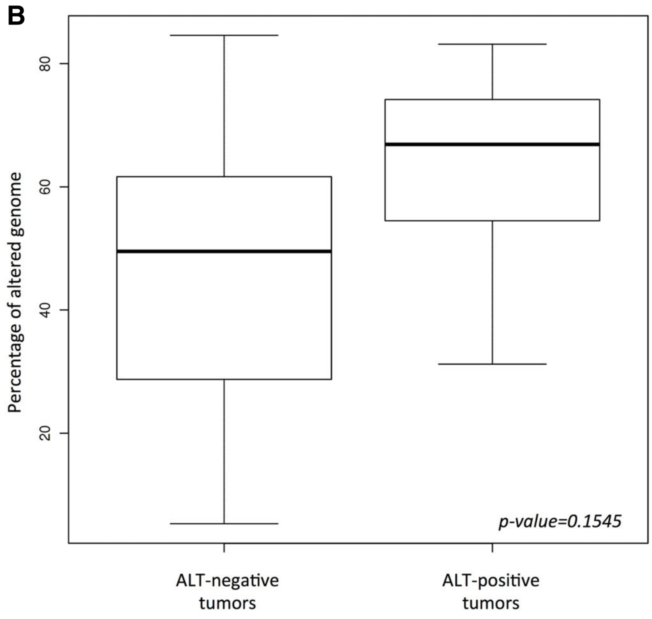

Figure EV2. No difference was observed in tumor mutational burden or percentage of altered genome according to ALT.

A Tumor mutational burden in ALT-negative (n = 6) and ALT-positive (n = 16) tumors. Box-and-whisker plot were defined with default parameters by median value (central band at the 50th percentile), interquartile ranges (IQR, box limited by 25th and 75th percentile) and whisker boundaries defined by minimum and maximum value. Wilcoxon test was used to compare modalities.

B Percentage of altered genome using array-comparative genomic hybridization (aCGH) in ALT-negative (n = 6) and ALT-positive (n = 16) tumors. Box-and-whisker plot were defined with default parameters by median value (central band at the 50th percentile), interquartile ranges (IQR, box limited by 25th and 75th percentile) and whisker boundaries defined by minimum and maximum value. Wilcoxon test was used to compare modalities.

 © 2022 The Authors

Figure EV3. Histone genes in patient's osteosarcoma tumors.

A Lollipop plots of *H1.4*, *H2A*, *H3.1*, and *H4* genes showing histone mutations detected by tNGS.

B Expression of histone genes according to ALT and ATRX status: $ALT^{+}/ATRX^{+}$ (n = 7), $ALT^{+}/ATRX^{-}$ (n = 2) et ALT^{-} (n = 3). Box-and-whisker plot were defined with default parameters by median value (central band at the 50th percentile), and interquartile ranges (IQR, box limited by 25th and 75th percentile) and whisker boundaries were defined by minimum and maximum value. An ANOVA statistical test, and Tukey's range test were used to compare modalities.

© 2022 The Authors

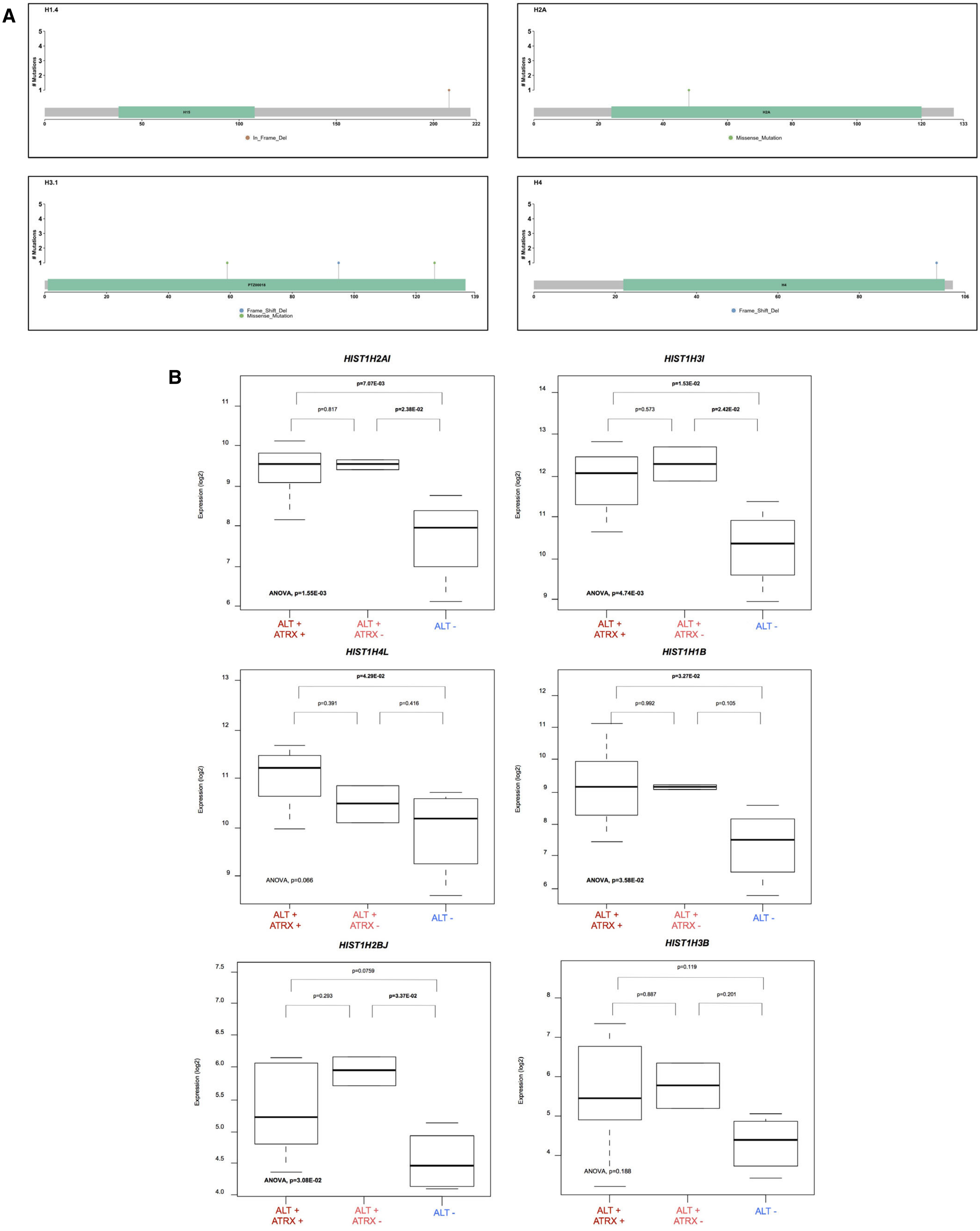

Figure EV3.

 © 2022 The Authors

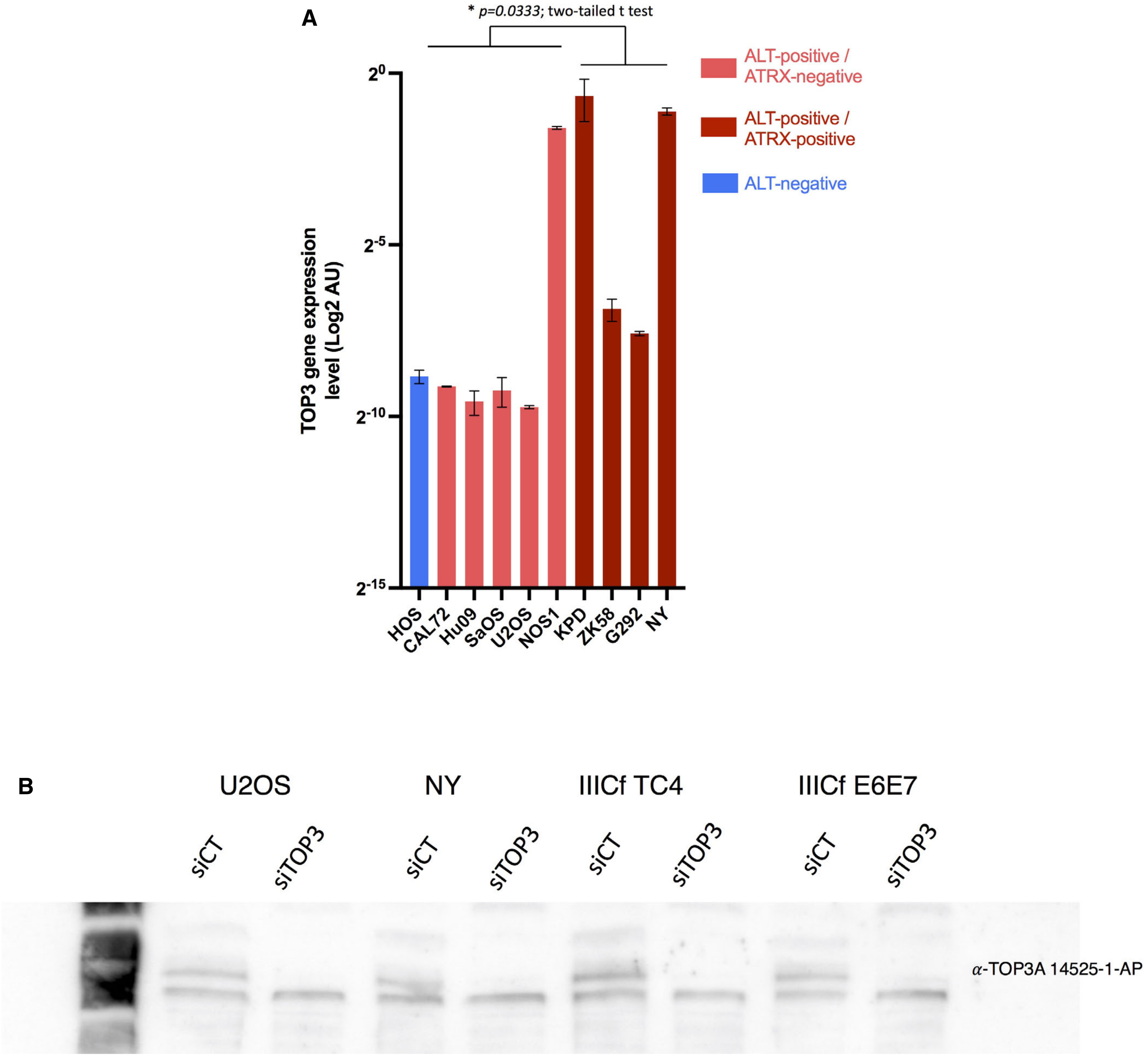

Figure EV4. TOP3A expression in osteosarcoma cell lines.

A TOP3A expression (by RT-qPCR) in human osteosarcoma cell lines. Mean expression level of three technical replicates; error bars represent the mean ± SEM.

B TOP3A Western blots validating siRNA knockdowns in the indicated cell lines (U2OS, NY, IIIcf TC4, IIICf E6E7).

© 2022 The Authors

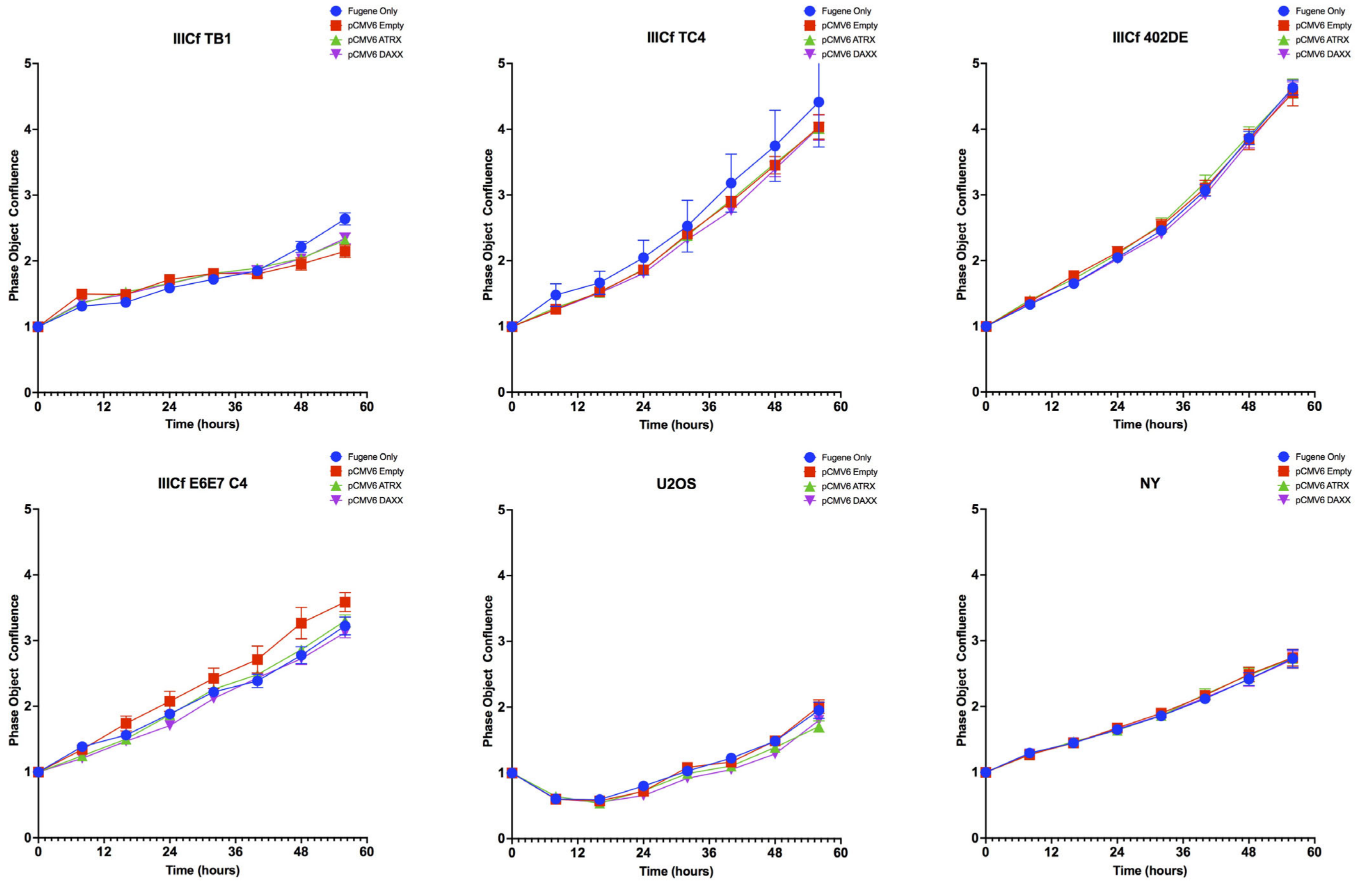

Figure EV5. Effect of ectopic ATRX expression.

Growth curve (Points and error bars (±SEM) represent extrapolation of phase-contrast object confluence assessed by Incucyte optical system from $n = 3$ technical replicates) according to transfection condition (FuGENE only, pCMV6-empty, –ATRX, -DAXX).

 © 2022 The Authors
