# Supplementary material for: TOP3A amplification and ATRX inactivation are mutually exclusive events in pediatric osteosarcomas using ALT
Source: EMBO Mol Med. 2022 Aug 3;14(10):e15859. doi: 10.15252/emmm.202215859 (PMC9549729; doi:10.15252/emmm.202215859)
Supplement: Supplementary file 7 [file EMMM-14-e15859-s007.pdf]

EMBO Molecular Medicine

TOP3A amplification and ATRX inactivation are mutually exclusive events in pediatric osteosarcomas using ALT

Alexandre de Nonneville, Sebastien Salas, François Bertucci, Alexander Sobinoff, José Adélaide, Arnaud GUILLE, Pascal Finetti, Jane Noble, Dmitri Churikov, Max Chaffanet, Elise Lavit, Hilda Pickett, Corinne Bouvier, Daniel Birnbaum, Roger Reddel, and Vincent Geli
DOI: 10.15252/emmm.202215859

Corresponding authors: Vincent Geli (vincent.geli@inserm.fr) , Roger Reddel (rreddel@cmri.org.au), Daniel Birnbaum (daniel.birnbaum@inserm.fr)

Review Timeline:

Submission Date:	9th Feb 22
Editorial Decision:	3rd Mar 22
Revision Received:	4th Jul 22
Editorial Decision:	11th Jul 22
Revision Received:	13th Jul 22
Accepted:	14th Jul 22

Editor: Lise Roth

Transaction Report:

(Note: With the exception of the correction of typographical or spelling errors that could be a source of ambiguity, letters and reports are not edited. Depending on transfer agreements, referee reports obtained elsewhere may or may not be included in this compilation. Referee reports are anonymous unless the Referee chooses to sign their reports.)

3rd Mar 2022

Dear Dr. Geli,

Thank you for the submission of your manuscript to EMBO Molecular Medicine. We have now received feedback from the three reviewers who agreed to evaluate your manuscript. As you will see from the reports below, the referees acknowledge the interest of the study and are overall supporting publication of your work pending appropriate revisions.

Addressing the reviewers' concerns in full will be necessary for further considering the manuscript in our journal, and acceptance of the manuscript will entail a second round of review. EMBO Molecular Medicine encourages a single round of revision only and therefore, acceptance or rejection of the manuscript will depend on the completeness of your responses included in the next, final version of the manuscript. For this reason, and to save you from any frustrations in the end, I would strongly advise against returning an incomplete revision.

Revised manuscripts should be submitted within three months of a request for revision; they will otherwise be treated as new submissions, except under exceptional circumstances in which a short extension is obtained from the editor.

When submitting your revised manuscript, please carefully review the instructions that follow below. We perform an initial quality control of all revised manuscripts before re-review; failure to include requested items will delay the evaluation of your revision.

We require:

1) A .docx formatted version of the manuscript text (including legends for main figures, EV figures and tables). Please make sure that the changes are highlighted to be clearly visible.

2) Individual production quality figure files as .eps, .tif, .jpg (one file per figure). For guidance, download the 'Figure Guide PDF' (https://www.embopress.org/page/journal/17574684/authorguide#figureformat).

3) A .docx formatted letter INCLUDING the reviewers' reports and your detailed point-by-point responses to their comments. As part of the EMBO Press transparent editorial process, the point-by-point response is part of the Review Process File (RPF), which will be published alongside your paper.

4) A complete author checklist, which you can download from our author guidelines (https://www.embopress.org/page/journal/17574684/authorguide#submissionofrevisions). Please insert information in the checklist that is also reflected in the manuscript. The completed author checklist will also be part of the RPF.

5) It is mandatory to include a 'Data Availability' section after the Materials and Methods. Before submitting your revision, primary datasets produced in this study need to be deposited in an appropriate public database, and the accession numbers and database listed under 'Data Availability'. Please remember to provide a reviewer password if the datasets are not yet public (see https://www.embopress.org/page/journal/17574684/authorguide#dataavailability).
In case you have no data that requires deposition in a public database, please state so in this section. Note that the Data Availability Section is restricted to new primary data that are part of this study.

6) For data quantification: please specify the name of the statistical test used to generate error bars and P values, the number (n) of independent experiments (specify technical or biological replicates) underlying each data point and the test used to calculate p-values in each figure legend. The figure legends should contain a basic description of n, P and the test applied. Graphs must include a description of the bars and the error bars (s.d., s.e.m.). Exact p values should be provided.

7) We would also encourage you to include the source data for figure panels that show essential data. Numerical data should be provided as individual .xls or .csv files (including a tab describing the data). For blots or microscopy, uncropped images should be submitted (using a zip archive if multiple images need to be supplied for one panel). Additional information on source data and instruction on how to label the files are available at
.

8) Our journal encourages inclusion of *data citations in the reference list* to directly cite datasets that were re-used and obtained from public databases. Data citations in the article text are distinct from normal bibliographical citations and should directly link to the database records from which the data can be accessed. In the main text, data citations are formatted as follows: "Data ref: Smith et al, 2001" or "Data ref: NCBI Sequence Read Archive PRJNA342805, 2017". In the Reference list, data citations must be labeled with "[DATASET]". A data reference must provide the database name, accession number/identifiers and a resolvable link to the landing page from which the data can be accessed at the end of the reference. Further instructions are available at .

9) We replaced Supplementary Information with Expanded View (EV) Figures and Tables that are collapsible/expandable online. A maximum of 5 EV Figures can be typeset. EV Figures should be cited as 'Figure EV1, Figure EV2" etc... in the text and their respective legends should be included in the main text after the legends of regular figures.
- For the figures that you do NOT wish to display as Expanded View figures, they should be bundled together with their legends in a single PDF file called *Appendix*, which should start with a short Table of Content. Appendix figures should be referred to in the main text as: "Appendix Figure S1, Appendix Figure S2" etc.
- Additional Tables/Datasets should be labeled and referred to as Table EV1, Dataset EV1, etc. Legends have to be provided in a separate tab in case of .xls files. Alternatively, the legend can be supplied as a separate text file (README) and zipped together with the Table/Dataset file.
See detailed instructions here:
.

10) The paper explained: EMBO Molecular Medicine articles are accompanied by a summary of the articles to emphasize the major findings in the paper and their medical implications for the non-specialist reader. Please provide a draft summary of your article highlighting
- the medical issue you are addressing,
- the results obtained and
- their clinical impact.

This may be edited to ensure that readers understand the significance and context of the research. Please refer to any of our published articles for an example.

11) For more information: There is space at the end of each article to list relevant web links for further consultation by our readers. Could you identify some relevant ones and provide such information as well? Some examples are patient associations, relevant databases, OMIM/proteins/genes links, author's websites, etc...

12) Conflict of interest: We updated our journal's competing interests policy in January 2022 and request authors to consider both actual and perceived competing interests. Please review the policy https://www.embopress.org/competing-interests and update your competing interests if necessary.

13) Every published paper now includes a 'Synopsis' to further enhance discoverability. Synopses are displayed on the journal webpage and are freely accessible to all readers. They include a short stand first (maximum of 300 characters, including space) as well as 2-5 one-sentences bullet points that summarizes the paper. Please write the bullet points to summarize the key NEW findings. They should be designed to be complementary to the abstract - i.e. not repeat the same text. We encourage inclusion of key acronyms and quantitative information (maximum of 30 words / bullet point). Please use the passive voice. Please attach these in a separate file or send them by email, we will incorporate them accordingly.
Please also suggest a striking image or visual abstract to illustrate your article as a PNG file 550 px wide x 300-600 px high.

14) As part of the EMBO Publications transparent editorial process initiative (see our Editorial at http://embomolmed.embopress.org/content/2/9/329), EMBO Molecular Medicine will publish online a Review Process File (RPF) to accompany accepted manuscripts.
In the event of acceptance, this file will be published in conjunction with your paper and will include the anonymous referee reports, your point-by-point response and all pertinent correspondence relating to the manuscript. Let us know whether you agree with the publication of the RPF and as here, if you want to remove or not any figures from it prior to publication.
Please note that the Authors checklist will be published at the end of the RPF.

EMBO Molecular Medicine has a "scooping protection" policy, whereby similar findings that are published by others during review or revision are not a criterion for rejection. Should you decide to submit a revised version, I do ask that you get in touch after three months if you have not completed it, to update us on the status.

I look forward to receiving your revised manuscript.

Yours sincerely,

Lise Roth

Lise Roth, PhD
Editor
EMBO Molecular Medicine

***** Reviewer's comments *****

Referee #1 (Remarks for Author):

In this report by de Nonneville A et al., the authors investigated the frequency of ALT in high-grade osteosarcomas from 22 non-metastatic and non-pre-treated pediatric patients. They found that 73% of the samples showed hallmarks of ALT. Patients with ALT-positive tumors were more prone to relapse and experience poor disease-free survival rates. Targeted sequencing approaches revealed that the TOP3A gene is frequently amplified in ALT+ tumors. From these studies, the authors also performed RNA-sequencing on 9 ALT+ tumors and 3 ALT- tumors and found TOP3A among other genes that are upregulated in ALT+ tumors. The authors found that among 9 ALT+ osteosarcoma cell lines, the 4 cell lines that showed the presence of wildtype ATRX protein, showed higher expression of TOP3a. Interestingly, the recruitment of TOP3A to telomeres is not affected by ATRX status. Moreover, the frequency of TIFs or APBs was similarly affected by TOP3A depletion in both ATRX+ or ATRX- cells. This is a nice study. However, several important experiments are needed to support the claims.

Strengths:

• The authors find that the TOP3A gene, among others, is highly expressed in ALT high-grade pediatric osteosarcomas.
• This study puts forth the notion that there may be an independent mechanism of ALT activation that requires TOP3A activation, in an ATRX-independent manner.
• The performed experiments have appropriate controls and are well performed.

Limitations:
• While the results are quite interesting, the authors use targeted NGS of 756 genes and not exome sequencing to find genes that are differently mutated in ALT+ vs ALT- tumors. This may have eliminated other genes that may contribute to this variation.
• The TOP3a amplified region on 17p11 is quite close to the p53 locus (17p13). Several associations are shown (ATRX, gender, stage etc) but not p53 status. Could this be provided?
• The expression of TOP3a mRNA in the ATRX WT ALT+ cell lines was modestly elevated compared to ATRXmut ALT+ cell lines. Also, no western blot was shown. This should be provided. Similarly, validation of siRNA knockdown in each of the necessary cell lines and conditions is not provided.
• The test of functional ATRX in the ALT+ATRX+ cell lines involved ATRX overexpression instead of a functional assay such as an immunofluorescence-based approach to observe the localization of ATRX to telomeres. No western is shown either.
• The authors do not address why TOP3a depletion does not affect ALT hallmarks like APBs in the ATRX WT ALT- cell lines through extensive experimentation. It remains unclear what the consequence of TOP3A amplification in these cells is, and why it must not coexist with ATRX mutations. In fact, the authors could generate ATRX KO cells in the cell lines that are ALT- TOP3A amplified ATRX WT and ask if this impacts cell survival. Similarly, the authors could compare the effects on survival of TOP3a depletion on ATRX+ vs ATRX- ALT and non-ALT cell lines.
• It is also unclear what the mechanism of DNA synthesis is in TOP3a amplification cells? Since APBs are unaffected and these are believed to represent sites of RAD52/RAD51 dependent DNA synthesis (Zhang et al., Cell Reports) - then which pathway is TOP3A functioning in. For example, as the authors mention TOP3A being a member of the BTR complex, if the overexpression of TOP3A yields to more BLM localization at ALT telomeres should be investigated and is not beyond the scope of the study.

Minor Issues: check the spelling on 5E y axis.

Additionally.

This reviewer finds that the authors are pushing needlessly hard on the idea that ATRX mutation and loss of function/expression is NOT ALT. It seems to me that this issue was arbitrated previously in the "matters arising" in Nature Communications. The authors state "ATRX loss has been proposed as a marker of ALT (Sieverling et al, 2020), but the view that ATRX/DAXX loss is essentially equivalent to the presence of ALT activity may apply only to specific types of tumors, in specific genomic or epigenetic contexts, and may divert attention from the need to identify alternative molecular actors involved in this telomere maintenance mechanism (TMM) (Barthel et al, 2017; Brosnan-Cashman et al, 2018; Graham et al, 2019; de Nonneville & Reddel, 2021)" . I believe this is unwarranted. They state "may apply only to specific types of tumors" and I do not think that the Sieverling paper implied anything different to this. I think the authors should reconsider going after this so aggressively as their study is confined to a single tumor type with different properties.

Referee #2 (Remarks for Author):

Human cancer cells maintain telomeres by either telomerase or HR-based alternative lengthening of telomeres (ALT). Mounting evidence support the notion that ATRX mutation and/or loss of protein expression contribute to ALT. However, ATRX deficiency is detected in only 30% of ALT-positive high-grade osteosarcomas, suggesting that other pathways are responsible for the ALT mechanism in high-grade osteosarcomas. In this study, the authors identified amplification and overexpression of a number of genes, including TOP3A in pediatric high-grade osteosarcomas. TOP3A has been shown to be localized at telomeres in ALT cancer lines and promote telomere maintenance through several means, including POLD3-dependent telomere synthesis and mitotic DNA synthesis. Interestingly, TOP3A amplification and overexpression and ATRX mutations are mutually exclusive events in high-grade osteosarcoma. The authors further demonstrated that overexpression of ectopic TOP3A is sufficient to promote ALT hallmarks, such as C-circles and APBs in ATXR-deficient ALT cancer cells expressing ectopic ATRX. In addition, TO3A knock-down decreases C-circle in ATRX-proficient ALT cancer cells. these data demonstrate that TOP3A is an ALT regulator in the ATRX-positive setting. This study developed a novel ALT hallmark and identified that ATRX inactivation or TOP3A amplification may represent two independent ways of promoting ALT. The experiments are well designed and executed. The manuscript was well written. Some data presentation needs further clarification.

1. The authors have shown that TOP3A knock-down led to the appearance of very short telomeres in ALT-positive ATRX-wt cell lines? Typical ALT cancer lines show telomere length heterogeneity, with very long and short telomeres. Thus, very short telomeres may not affect ALT cancer viability. Does TOP3A knock-down affect the growth rate in any of the ALT-positive ATRX-wt cell lines, such as (IIICF DE, IIICF E6E7, and NY? This experiment will further strengthen the role of TOP3A in ALT-positive ATRX-wt cancers.

2. Supplementary figure 5A, please fix the alignment for pCMV/6 DAXX

3. Title: please replace "pediatric OS" with "pediatric osteosarcomas".

Referee #3 (Comments on Novelty/Model System for Author):

The work is well done, has sufficient sample numbers and is clear and considered in the interpretation. Overall it is a very good standard study using primary human OS with sufficient validation and experimental confirmation.

Referee #3 (Remarks for Author):

The manuscript by de Nonneville et al assessed the association of ALT and ATRX in primary human osteosarcoma sample. They determine that TOP3A was overexpressed (due to genomic amplification) in the ALT-positive ATRX-wt tumors. Using knock-down and overexpression that demonstrate that targeting TOP3A can alter the ALT state in ATRX wt cells and that this may be a therapeutic target/stratification tool.

Overall the paper is well written, accessible and understandable and the figures are well presented. The interpretation is appropriate for the results presented and the discussion is detailed and balanced.
I do not have any useful comments or additional experiments that I can think of that would improve the work or challenge any of the findings.

Referee #1 (Remarks for Author):

1) The TOP3a amplified region on 17p11 is quite close to the p53 locus (17p13). Several associations are shown (ATRX, gender, stage etc) but not p53 status. Could this be provided?

We thank referee 1 for this remark. The TP53 status is now provided in a new table of the manuscript (Table EV5, page 9).
We found that in most tumors in which TOP3A is amplified (4001, 4078, 4591, 4665, 5136), TP53 is at the telomeric edge of the TOP3A amplification. The TP53 locus may be disrupted (See the new aCGH Figure 3C). Although the TP53 gene is only represented on the aCGH chip by two oligos, it seems likely that amplification leads to the inactivation of this allele of TP53 followed by amplification of the centromeric region containing TOP3A. This raises the interesting hypothesis that amplification of this genomic region could do "double duty", on the one hand amplifying TOP3A and on the other hand inactivating TP53.

2) The expression of TOP3a mRNA in the ATRX WT ALT+ cell lines was modestly elevated compared to ATRXmut ALT+ cell lines. Also, no western blot was shown. This should be provided.

As asked by Referee 1, we provided in the revised version a TOP3A Western run with the samples shown in Fig. 5A corresponding to the ATRX Western blot. This new panel (Fig. 5B in the revised version, page 11) reveals that over-expression of TOP3A correlates with the presence of ATRX in cell lines (except for the KPD cell line).

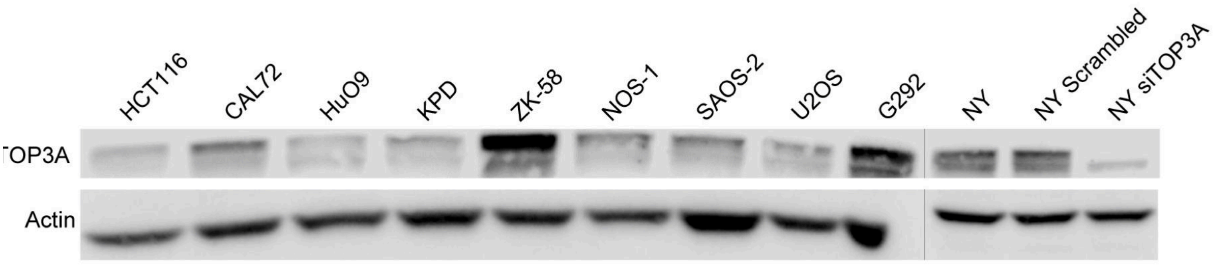

3) Validation of siRNA knockdown in each of the necessary cell lines and conditions is not provided.
We provided in the revised version the TOP3A Western blots validating the siRNA knockdowns in the cell lines (U2OS, NY, IIIcf TC4, IIICf E6E7) (Fig EV4B).

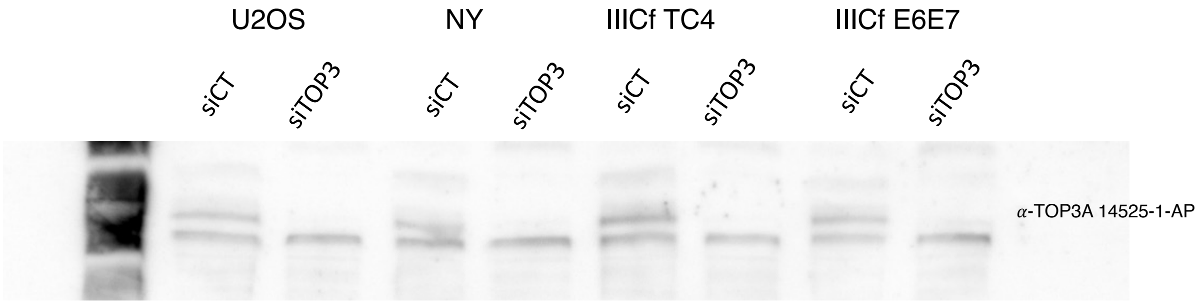

The conditions are now provided in Materials and Methods: two TOP3A Silencer Select siRNAs were designed and synthesized by Life Technologies (s14311 & s224746), and Silencer Select RNAi siRNA Negative Control #2 (#4390847) was used as the control siRNA. Cell suspensions were transfected at 20–50 % confluency with Lipofectamine RNAiMAX (Life Technologies) at a final siRNA concentration of 30 nM. Culture medium was changed after 48 h and cells were harvested for analysis 72 h post-transfection. Knockdown efficiency was validated by western blot analysis.

4) The test of functional ATRX in the ALT+ATRX+ cell lines involved ATRX overexpression instead of a functional assay such as an immunofluorescence-based approach to observe the localization of ATRX to telomeres. No western is shown either.

We provide in the "Appendix" a representative IF experiment showing the localization of ATRX to telomeres in ALT+ATRX+ cell lines (NY cell line).

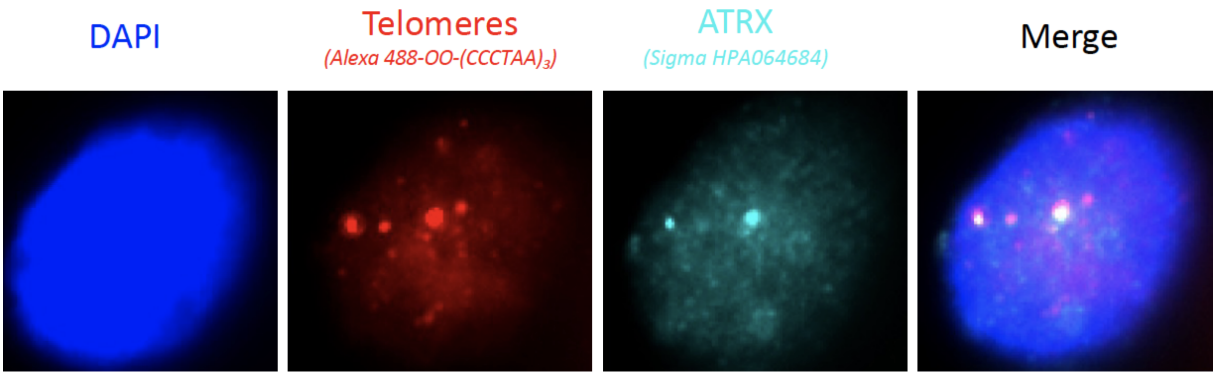

5) The authors do not address why TOP3a depletion does not affect ALT hallmarks like APBs in the ATRX WT ALT- cell lines through extensive experimentation.

This point raised by the referee is not entirely clear to us. Does the referee refer to ATRX WT ALT- (ALT negative) cell lines or to ATRX WT ALT positive cell lines? TOP3A KD affects ALT hallmarks in ATRX WT ALT positive cell lines.

As shown below in the new Figure 7, TOP3A depletion affects BLM localization at APBs and ALT DNA synthesis in both ALT cell lines U2OS (ATRX mut) and NY (ATRX wt), but the effect of TOP3A deletion is much more pronounced in the NY cell line that overexpresses TOP3A.

6) It remains unclear what the consequence of TOP3A amplification in these cells is, and why it must not coexist with ATRX mutations.

They are most likely mutually exclusive because 1 pathway (TOP3A amplification or ATRX inactivation) is enough to ensure the clonal selection of ALT cancer cells.

7) The authors could generate ATRX KO cells in the cell lines that are ALT- TOP3A amplified ATRX WT and ask if this impacts cell survival. Similarly, the authors could compare the effects on survival of TOP3a depletion on ATRX+ vs ATRX- ALT and non-ALT cell lines

We regard deletion of ATRX in cells amplified for TOP3A for the reasons discussed in point 6.

8) It is also unclear what the mechanism of DNA synthesis is in TOP3a amplification cells? Since APBs are unaffected and these are believed to represent sites of RAD52/RAD51 dependent DNA synthesis (Zhang et al., Cell Reports) - then which pathway is TOP3A functioning in.

9) The authors mention TOP3A being a member of the BTR complex, if the overexpression of TOP3A yields to more BLM localization at ALT telomeres should be investigated and is not beyond the scope of the study.

To first answer point 9, we monitored the localization of BLM in U2OS and NY cell lines. As pointed by referee 1, because NY cells overexpress *TOP3A*, this should lead to more BLM at ALT telomeres. As shown in the new Figure 7A (see below), we observed an increase in the intensity of BLM at telomeres in the NY cells compared to U2OS cells. We also found that TOP3A depletion clearly affects BLM localization at APBs in both cell lines.

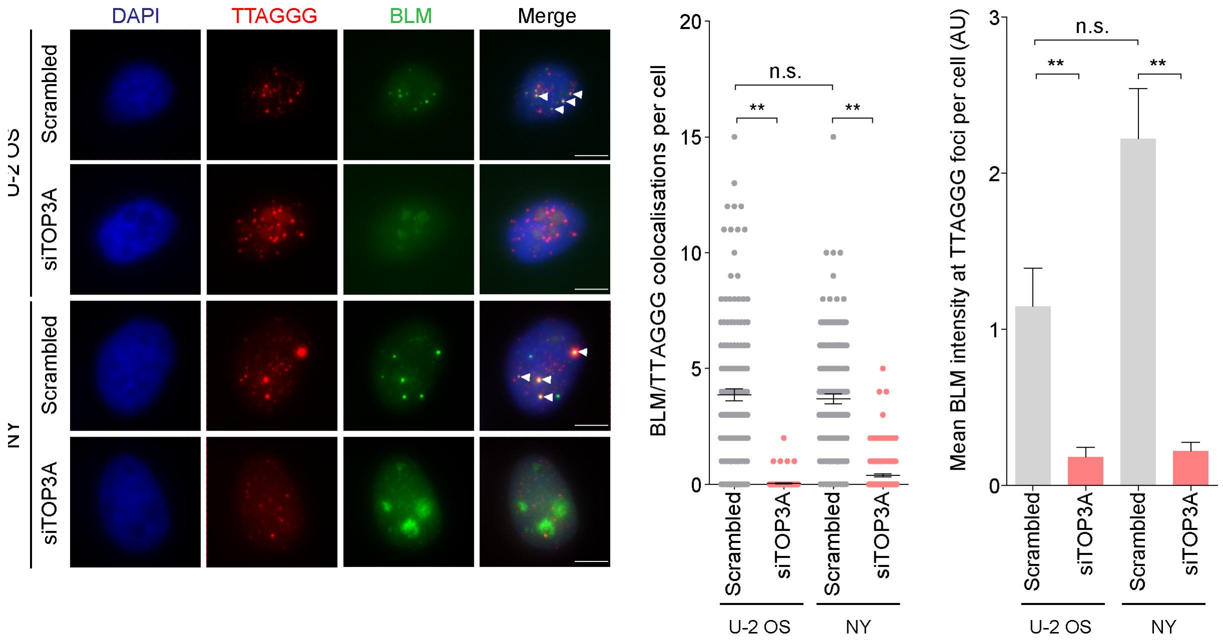

Given this result, we performed an ATSA Assay (Sobinoff et al., 2017, Zhang et al. 2018) to test whether TOP3A KD disrupts telomeric DNA synthesis in the two cell lines U2OS (ATRX-negative) and NY (ATRX-positive). We found that the NY cell line has higher levels of ALT DNA synthesis and that TOP3A depletion compromises ALT DNA synthesis in both cell lines (new Figure 7B here below).

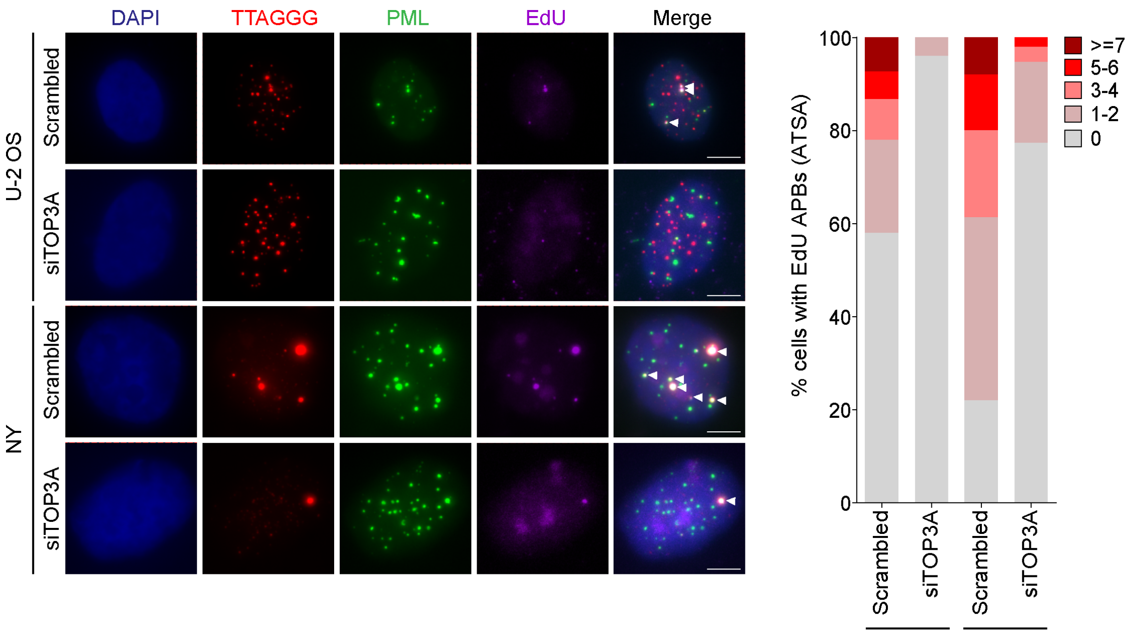

Taken together, these results indicate that increased levels of TOP3A in NY cells correlate with increased levels of BLM at ALT telomeres and that TOP3A promotes ALT DNA synthesis. These results are consistent with the greater dependence of NY cells on TOP3A.

This new Figure 7 (panels A and B) was performed by Alexander Sobinoff and Hilda Pickett who were added as new co-authors to the manuscript. The experimental procedures of these two experiments are described in Materials and methods.

Minor Issues: check the spelling on 5E y axis.
Done

Additionally.

This reviewer finds that the authors are pushing needlessly hard on the idea that ATRX mutation and loss of function/expression is NOT ALT. It seems to me that this issue was arbitrated previously in the "matters arising" in Nature Communications. The authors state "ATRX loss has been proposed as a marker of ALT (Sieverling et al, 2020), but the view that ATRX/DAXX loss is essentially equivalent to the presence of ALT activity may apply only to specific types of tumors, in specific genomic or epigenetic contexts, and may divert attention from the need to identify alternative molecular actors involved in this telomere maintenance mechanism (TMM) (Barthel et al, 2017; Brosnan-Cashman et al, 2018; Graham et al, 2019; de Nonneville & Reddel, 2021)" . I believe this is unwarranted. They state "may apply only to specific types of tumors" and I do not think that the Sieverling paper implied anything different to this. I think the authors should reconsider going after this so aggressively as their study is confined to a single tumor type with different properties.

We have modified the wording as requested by the reviewer.

Referee #2 (Remarks for Author):

Human cancer cells maintain telomeres by either telomerase or HR-based alternative lengthening of telomeres (ALT). Mounting evidence support the notion that ATRX mutation

and/or loss of protein expression contribute to ALT. However, ATRX deficiency is detected in only 30% of ALT-positive high-grade osteosarcomas, suggesting that other pathways are responsible for the ALT mechanism in high-grade osteosarcomas. In this study, the authors identified amplification and overexpression of a number of genes, including TOP3A in pediatric high-grade osteosarcomas. TOP3A has been shown to be localized at telomeres in ALT cancer lines and promote telomere maintenance through several means, including POLD3-dependent telomere synthesis and mitotic DNA synthesis. Interestingly, TOP3A amplification and overexpression and ATRX mutations are mutually exclusive events in high-grade osteosarcoma. The authors further demonstrated that overexpression of ectopic TOP3A is sufficient to promote ALT hallmarks, such as C-circles and APBs in ATXR-deficient ALT cancer cells expressing ectopic ATRX. In addition, TO3A knock-down decreases C-circle in ATRX-proficient ALT cancer cells. these data demonstrate that TOP3A is an ALT regulator in the ATRX-positive setting. This study developed a novel ALT hallmark and identified that ATRX inactivation or TOP3A amplification may represent two independent ways of promoting ALT. The experiments are well designed and executed. The manuscript was well written. Some data presentation needs further clarification.

We thank Referee 2 for his/her general comments.

1. The authors have shown that TOP3A knock-down led to the appearance of very short telomeres in ALT-positive ATRX-wt cell lines? Typical ALT cancer lines show telomere length heterogeneity, with very long and short telomeres.
Thus, very short telomeres may not affect ALT cancer viability. Does TOP3A knock-down affect the growth rate in any of the ALT-positive ATRX-wt cell lines, such as (IIICF DE, IIICF E6E7, and NY? This experiment will further strengthen the role of TOP3A in ALT-positive ATRX-wt cancers.

We have monitored the growth rate in a number of ATRX wt cell lines and did not observe any differences in the growth rate of these cell lines upon TOP3A depletion. Unfortunately, we did not monitor the growth rate of the NY cells upon TOP3A depletion. However as mentioned above, we provide new experiments (Figure 7) that strengthen the role of TOP3A in the ALT ATRX-wt NY cells.

2. Supplementary figure 5A, please fix the alignment for pCMV/6 DAXX
Done

3. Title: please replace "pediatric OS" with "pediatric osteosarcomas".
Done

Referee #3 (Comments on Novelty/Model System for Author):

The work is well done, has sufficient sample numbers and is clear and considered in the interpretation. Overall it is a very good standard study using primary human OS with sufficient validation and experimental confirmation.

We thank referee 3 for these comments.

Referee #3 (Remarks for Author):

The manuscript by de Nonneville et al assessed the association of ALT and ATRX in primary human osteosarcoma sample. They determine that TOP3A was overexpressed (due to genomic amplification) in the ALT-positive ATRX-wt tumors. Using knock-down and overexpression that demonstrate that targeting TOP3A can alter the ALT state in ATRX wt cells and that this may be a therapeutic target/stratification tool.

Overall the paper is well written, accessible and understandable and the figures are well presented. The interpretation is appropriate for the results presented and the discussion is detailed and balanced.
I do not have any useful comments or additional experiments that I can think of that would improve the work or challenge any of the findings.

We thank referee 3 for these comments.

11th Jul 2022

Dear Vincent,

Thank you for the submission of your manuscript to EMBO Molecular Medicine. We have received the enclosed report from referee #1 who re-reviewed your manuscript. As you will see, this referee is now supportive of publication, and I am therefore pleased to inform you that we will be able to accept your manuscript once the following editorial points will be addressed:

1/ Main manuscript text:
- Please remove the red text, accept the changes and only keep in track changes mode any new modification.
- We note (and you informed us in your cover letter) that you currently have together with you a total of 3 co-corresponding authors. Is that correct? Do you confirm equal contribution of these 3 authors, able to take full responsibility for the paper and its content? While there is no limit per se to the number of co-corresponding authors, 3 is rare and may not reflect as intended to the community.
- Material and Methods:
o Patients' samples/Ethics Declaration: Please include a statement that the experiments conformed to the principles set out in the WMA Declaration of Helsinki and the Department of Health and Human Services Belmont Report.
o Western blots: please indicate the dilutions used for primary antibodies.
- Thank you for providing a Data Availability Section. Please note that the data must be made public before acceptance of the manuscript.

2/ Figures:
Please indicate in the figures or in their legends the exact p values, not a range (including for non significant (n.s.) p values). Some authors prefer to add a table in the appendix to keep their figures clear, you are welcome to do this if you wish.

3/ Thank you for providing the Paper Explained. I added minor modifications in the text, please let me know if you agree with the following, or amend as you see fit:
Medical issue
To maintain their telomeres, most human cancer cells upregulate telomerase, while the rest uses a mechanism based on homologous recombination-mediated DNA replication called alternative lengthening of telomeres (ALT). High-grade osteosarcomas are highly aggressive bone tumors that mainly occur in children and adolescents, with limited treatment options. Strikingly, osteosarcomas exhibit a high frequency of ALT. In contrast to many other tumors, ATRX, a chromatin remodeler, is mutated in only 30 % of ALT-positive osteosarcomas, indicating that these may not be the only alterations responsible for ALT.
Results
We characterized non-metastatic, non-pre-treated, high-grade pediatric osteosarcomas and uncovered that more than 70 % of these tumors maintained their telomeres via the ALT pathway. In the ALT tumors, TOP3A gene amplification and overexpression was more prevalent than ATRX loss-of-function. Because ATRX mutation and TOP3A overexpression were mutually exclusive, overexpression of TOP3A appeared as an alternative mechanism driving ALT in pediatric osteosarcoma. In ALT-positive human osteosarcoma-derived cell lines, TOP3 overexpression also correlated with the presence of ATRX. Moreover, TOP3A was required to localize the BLM helicase to telomeres and promote ALT-associated DNA synthesis. Several genes whose alteration is associated with ALT in pediatric osteosarcoma were additionally identified, revealing new mechanistic insights into telomere maintenance in these tumors. Overall, these results identify TOP3A or TOP3A downstream signaling as a potential therapeutic target.
Impact
Amplification of TOP3A is a new hallmark of the ALT mechanism in tumors that are otherwise wild-type for ATRX. The discovery that TOP3A amplification/overexpression is mutually exclusive with ATRX mutation constitutes a strong argument for the involvement of TOP3A in oncogenesis. Our work therefore positions TOP3A action at telomeres as a prognostic marker and a new potential therapeutic target in ALT-associated pediatric osteosarcomas.

4/ I also slightly edited your synopsis, please let me know if you agree with the following:
There is an urgent need to treat pediatric osteosarcomas that present high frequency of Alternative Lengthening of Telomeres (ALT). This study identifies TOP3A amplification as mutually exclusive with ATRX inactivation, promoting ALT and TOP3A action at telomeres as a novel therapeutic target.
- 17p11 amplification and ATRX mutation are exclusive genomic events in ALT high-grade pediatric osteosarcomas
- TOP3A overexpression is a feature of ATRX-wt ALT osteosarcomas
- Over-expression of TOP3A correlates with the presence of ATRX in ATRX-wt ALT cell lines
- TOP3A is required for proper BLM localization in ALT osteosarcoma cell lines and promotes ALT-mediated telomeric DNA synthesis

5/ As part of the EMBO Publications transparent editorial process initiative (see our Editorial at

http://embomolmed.embopress.org/content/2/9/329), EMBO Molecular Medicine will publish online a Review Process File (RPF) to accompany accepted manuscripts.
This file will be published in conjunction with your paper and will include the anonymous referee reports, your point-by-point response and all pertinent correspondence relating to the manuscript. Let us know whether you agree with the publication of the RPF and as here, if you want to remove or not any figures from it prior to publication.
Please note that the Authors checklist will be published at the end of the RPF.

I look forward to receiving your revised manuscript.

With kind regards,

Lise

Lise Roth, PhD
Senior Editor
EMBO Molecular Medicine

The system will prompt you to fill in your funding and payment information. This will allow Wiley to send you a quote for the article processing charge (APC) in case of acceptance. This quote takes into account any reduction or fee waivers that you may be eligible for. Authors do not need to pay any fees before their manuscript is accepted and transferred to our publisher.

***** Reviewer's comments *****

Referee #1 (Remarks for Author):

Thank you for addressing the comments. The paper is very nice.

The authors performed the requested editorial changes.

14th Jul 2022

Dear Vincent,

Thank you for submitting the revised files. I am pleased to inform you that your manuscript is accepted for publication and is now being sent to our publisher to be included in the next available issue of EMBO Molecular Medicine!

Please note that the datasets have to be public before online publication of the article (Link: https://www.ebi.ac.uk/arrayexpress/experiments/E-MTAB-11743 not yet accessible).

Please read below for additional IMPORTANT information regarding your article, its publication and the production process.

Congratulations on your interesting work!

With kind regards,

Lise

Lise Roth, Ph.D
Senior Editor
EMBO Molecular Medicine

Follow us on Twitter @EmboMolMed
Sign up for eTOCs at embopress.org/alertsfeeds

EMBO Press Author Checklist

Corresponding Author Name: Vincent Géli	
Journal Submitted to: EMM	
Manuscript Number: EMM-2022-15859	

USEFUL LINKS FOR COMPLETING THIS FORM
The EMBO Journal - Author Guidelines
EMBO Reports - Author Guidelines
Molecular Systems Biology - Author Guidelines
EMBO Molecular Medicine - Author Guidelines

Reporting Checklist for Life Science Articles (updated January 2022)
This checklist is adapted from Materials Design Analysis Reporting (MDAR) Checklist for Authors. MDAR establishes a minimum set of requirements in transparent reporting in the life sciences (see Statement of Task: 10.31222/osf.io/9sm4x). Please follow the journal's guidelines in preparing your manuscript.
Please note that a copy of this checklist will be published alongside your article.

Abridged guidelines for figures
1. Data
The data shown in figures should satisfy the following conditions:
- the data were obtained and processed according to the field's best practice and are presented to reflect the results of the experiments in an accurate and unbiased manner.
- ideally, figure panels should include only measurements that are directly comparable to each other and obtained with the same assay.
- plots include clearly labeled error bars for independent experiments and sample sizes. Unless justified, error bars should not be shown for technical replicates.
- if n<5, the individual data points from each experiment should be plotted. Any statistical test employed should be justified.
- Source Data should be included to report the data underlying figures according to the guidelines set out in the authorship guidelines on Data Presentation.

2. Captions
Each figure caption should contain the following information, for each panel where they are relevant:
- a specification of the experimental system investigated (eg cell line, species name).
- the assay(s) and method(s) used to carry out the reported observations and measurements.
- an explicit mention of the biological and chemical entity(ies) that are being measured.
- an explicit mention of the biological and chemical entity(ies) that are altered/varied/perturbed in a controlled manner.
- the exact sample size (n) for each experimental group/condition, given as a number, not a range;
- a description of the sample collection allowing the reader to understand whether the samples represent technical or biological replicates (including how many animals, litters, cultures, etc.).
- a statement of how many times the experiment shown was independently replicated in the laboratory.
- definitions of statistical methods and measures:
  - common tests, such as t-test (please specify whether paired vs. unpaired), simple χ2 tests, Wilcoxon and Mann-Whitney tests, can be unambiguously identified by name only, but more complex techniques should be described in the methods section;
  - are tests one-sided or two-sided?
  - are there adjustments for multiple comparisons?
  - exact statistical test results, e.g., P values = x but not P values < x;
  - definition of 'center values' as median or average;
  - definition of error bars as s.d. or s.e.m.

Please complete ALL of the questions below.
Select "Not Applicable" only when the requested information is not relevant for your study.

Materials

Newly Created Materials	Information included in the manuscript?	In which section is the information available? (Reagents and Tools Table, Materials and Methods, Figures, Data Availability Section)
New materials and reagents need to be available; do any restrictions apply?	Not Applicable	

Antibodies	Information included in the manuscript?	In which section is the information available? (Reagents and Tools Table, Materials and Methods, Figures, Data Availability Section)
For **antibodies** provide the following information:<br>- Commercial antibodies: RRID (if possible) or supplier name, catalogue number and or/clone number<br>- Non-commercial: RRID or citation	Yes	Materials and Methods

DNA and RNA sequences	Information included in the manuscript?	In which section is the information available? (Reagents and Tools Table, Materials and Methods, Figures, Data Availability Section)
**Short novel DNA or RNA including primers, probes**: provide the sequences.	Yes	Table EV8

Cell materials	Information included in the manuscript?	In which section is the information available? (Reagents and Tools Table, Materials and Methods, Figures, Data Availability Section)
**Cell lines:** Provide species information, strain. Provide accession number in repository **OR** supplier name, catalog number, clone number, and/**OR** RRID.	Yes	Materials and Methods
**Primary cultures:** Provide species, strain, sex of origin, genetic modification status.	Yes	Materials and Methods
Report if the cell lines were recently **authenticated** (e.g., by STR profiling) and tested for mycoplasma contamination.	Yes	Materials and Methods

Experimental animals	Information included in the manuscript?	In which section is the information available? (Reagents and Tools Table, Materials and Methods, Figures, Data Availability Section)
**Laboratory animals or Model organisms:** Provide species, strain, sex, age, genetic modification status. Provide accession number in repository **OR** supplier name, catalog number, clone number, **OR** RRID.	Not Applicable	
**Animal observed in or captured from the field:** Provide species, sex, and age where possible.	Not Applicable	
Please detail **housing and husbandry conditions**.	Not Applicable	

Plants and microbes	Information included in the manuscript?	In which section is the information available? (Reagents and Tools Table, Materials and Methods, Figures, Data Availability Section)
**Plants:** provide species and strain, ecotype and cultivar where relevant, unique accession number if available, and source (including location for collected wild specimens).	Not Applicable	
**Microbes:** provide species and strain, unique accession number if available, and source.	Not Applicable	

Human research participants	Information included in the manuscript?	In which section is the information available? (Reagents and Tools Table, Materials and Methods, Figures, Data Availability Section)
If collected and within the bounds of privacy constraints report on age, sex and gender or ethnicity for all study participants.	Yes	Table 1

Core facilities	Information included in the manuscript?	In which section is the information available? (Reagents and Tools Table, Materials and Methods, Figures, Data Availability Section)
If your work benefited from core facilities, was their service mentioned in the acknowledgments section?	Yes	Acknowledgements

Design

Study protocol	Information included in the manuscript?	In which section is the information available? (Reagents and Tools Table, Materials and Methods, Figures, Data Availability Section)
If study protocol has been **pre-registered, provide DOI in the manuscript**. For clinical trials, provide the trial registration number **OR** cite DOI.	Not Applicable	
Report the **clinical trial registration number** (at ClinicalTrials.gov or equivalent), where applicable.	Not Applicable	

Laboratory protocol	Information included in the manuscript?	In which section is the information available? (Reagents and Tools Table, Materials and Methods, Figures, Data Availability Section)
Provide DOI OR other citation details if **external detailed step-by-step protocols** are available.	Not Applicable	

Experimental study design and statistics	Information included in the manuscript?	In which section is the information available? (Reagents and Tools Table, Materials and Methods, Figures, Data Availability Section)
Include a statement about **sample size** estimate even if no statistical methods were used.	Yes	Materials and Methods
Were any steps taken to minimize the effects of subjective bias when allocating animals/samples to treatment (e.g. **randomization procedure**)? If yes, have they been described?	Not Applicable	
Include a statement about **blinding** even if no blinding was done.	Not Applicable	
Describe **inclusion/exclusion criteria** if samples or animals were excluded from the analysis. Were the criteria pre-established?<br><br>If sample or data points were omitted from analysis, report if this was due to attrition or intentional exclusion and provide justification.	Not Applicable	
For every figure, are **statistical tests** justified as appropriate? Do the data meet the assumptions of the tests (e.g., normal distribution)? Describe any methods used to assess it. Is there an estimate of variation within each group of data? Is the variance similar between the groups that are being statistically compared?	Yes	Materials and Methods

Sample definition and in-laboratory replication	Information included in the manuscript?	In which section is the information available? (Reagents and Tools Table, Materials and Methods, Figures, Data Availability Section)
In the figure legends: state number of times the experiment was **replicated** in laboratory.	Yes	Materials and Methods and Figure legends
In the figure legends: define whether data describe **technical or biological replicates**.	Yes	Figure legends

Ethics

Ethics	Information included in the manuscript?	In which section is the information available? (Reagents and Tools Table, Materials and Methods, Figures, Data Availability Section)
Studies involving **human participants**: State details of **authority granting ethics approval** (IRB or equivalent committee(s), provide reference number for approval.	Yes	Ethics Declaration
Studies involving **human participants**: Include a statement confirming that **informed consent** was obtained from all subjects and that the experiments conformed to the principles set out in the WMA Declaration of Helsinki and the Department of Health and Human Services Belmont Report.	Yes	Ethics Declaration
Studies involving **human participants:** For publication of **patient photos**, include a statement confirming that consent to publish was obtained.	Not Applicable	
Studies involving experimental **animals**: State details of **authority granting ethics approva**l (IRB or equivalent committee(s), provide reference number for approval. Include a statement of compliance with ethical regulations.	Not Applicable	
Studies involving **specimen and field samples:** State if relevant **permits** obtained, provide details of authority approving study; if none were required, explain why.	Not Applicable	

Dual Use Research of Concern (DURC)	Information included in the manuscript?	In which section is the information available? (Reagents and Tools Table, Materials and Methods, Figures, Data Availability Section)
Could your study fall under dual use research restrictions? Please check biosecurity documents and list of **select agents and toxins** (CDC): https://www.selectagents.gov/sat/list.htm	Not Applicable	
If you used a select agent, is the security level of the lab appropriate and reported in the manuscript?	Not Applicable	
If a study is subject to dual use research of concern regulations, is the name of the **authority granting approval and reference number** for the regulatory approval provided in the manuscript?	Not Applicable	

Reporting

The MDAR framework recommends adoption of discipline-specific guidelines, established and endorsed through community initiatives. Journals have their own policy about requiring specific guidelines and recommendations to complement MDAR.

Adherence to community standards	Information included in the manuscript?	In which section is the information available? (Reagents and Tools Table, Materials and Methods, Figures, Data Availability Section)
State if relevant guidelines or checklists (e.g., **ICMJE, MIBBI, ARRIVE, PRISMA**) have been followed or provided.	Not Applicable	
For **tumor marker prognostic studies,** we recommend that you follow the **REMARK** reporting guidelines (see link list at top right). See author guidelines, under 'Reporting Guidelines'. Please confirm you have followed these guidelines.	Not Applicable	
For **phase II and III randomized controlled trials**, please refer to the **CONSORT** flow diagram (see link list at top right) and submit the CONSORT checklist (see link list at top right) with your submission. See author guidelines, under 'Reporting Guidelines'. Please confirm you have submitted this list.	Not Applicable	

Data Availability

Data availability	Information included in the manuscript?	In which section is the information available? (Reagents and Tools Table, Materials and Methods, Figures, Data Availability Section)
Have **primary datasets** been deposited according to the journal's guidelines (see 'Data Deposition' section) and the respective accession numbers provided in the Data Availability Section?	Yes	Data availability
Were **human clinical and genomic datasets** deposited in a public access-controlled repository in accordance to ethical obligations to the patients and to the applicable consent agreement?	Yes	Data availability
Are **computational models** that are central and integral to a study available without restrictions in a machine-readable form? Were the relevant accession numbers or links provided?	Not Applicable	
If publicly available data were reused, provide the respective **data citations in the reference list**.	Not Applicable	
